# Preconception maternal gut dysbiosis affects enteric nervous system development and disease susceptibility in offspring via the GPR41–GDNF/RET/SOX10 signaling pathway

**DOI:** 10.1002/imt2.70012

**Published:** 2025-03-18

**Authors:** Cunzheng Zhang, Yuzhu Chen, Ruqiao Duan, Yiming Zhang, Haonan Zheng, Jindong Zhang, Tao Zhang, Jingxian Xu, Kailong Li, Fei Pei, Liping Duan

**Affiliations:** ^1^ Department of Gastroenterology Peking University Third Hospital Beijing China; ^2^ Beijing Key Laboratory for *Helicobacter pylori* Infection and Upper Gastrointestinal Diseases Beijing China; ^3^ PKUMed‐Wisbiom Joint Laboratory for Human Microbiome Research Beijing China; ^4^ Department of Biochemistry and Biophysics, Beijing Key Laboratory of Protein Posttranslational Modifications and Cell Function, School of Basic Medical Sciences Peking University Beijing China; ^5^ Department of Pathology Peking University Third Hospital Beijing China

**Keywords:** antibiotics, enteric nervous system, gut microbiota, offspring, preconception

## Abstract

Maternal health, specifically changes in the gut microbiota, can profoundly impact offspring health; however, our understanding of how gut microbiota alterations during the preconception period influence the offspring remains limited. In this study, we investigated the impact and mechanisms of preconception maternal gut dysbiosis on the development of the enteric nervous system (ENS) in mice. We found that preconception maternal exposure to antibiotics led to the abnormal development of the ENS in offspring, increasing their susceptibility to water avoidance stress at the adult stage. Metagenomic, targeted metabolomic, and transcriptomic analyses revealed that preconception antibiotic exposure disrupted the expression of genes crucial for embryonic ENS development by altering maternal gut microbiota composition. Multi‐omics analysis combined with *Limosilactobacillus reuteri* and propionate gestational supplementation demonstrated that the maternal gut microbiota and metabolites may influence embryonic ENS development via the GPR41–GDNF/RET/SOX10 signaling pathway. Our findings highlight the critical importance of maintaining a healthy maternal gut microbiota before conception to support normal ENS development in offspring.

## Highlights

- Preconception maternal antibiotic exposure results in the abnormal development of the enteric nervous system (ENS) and intestinal mucosa in mouse offspring, thereby increasing their susceptibility to water avoidance stress in adulthood.
- Preconception maternal antibiotic exposure alters the composition of the maternal gut microbiota, leading to abnormal embryonic ENS development via the modulation of gene expression.
- Multi‐omics analysis and supplementation with *Limosilactobacillus reuteri* and propionate during pregnancy indicate that the maternal gut microbiota and metabolites affect embryonic ENS development through the GPR41–GDNF/RET/SOX10 signaling pathway.

## Graphical abstract

Preconception administration of antibiotics to female mice reduces the abundance of *Limosilactobacillus reuteri* in the maternal gut microbiota during pregnancy, subsequently affecting propionate levels. Decreased propionate levels downregulated the expression of *Gdnf*/*Ret*/*Sox10* mediated by GPR41, leading to abnormal development of the enteric nervous system during the embryonic period. This dysplasia results in colonic dysmotility, impaired colonic epithelium, and increased susceptibility to water‐avoidance stress in offspring.

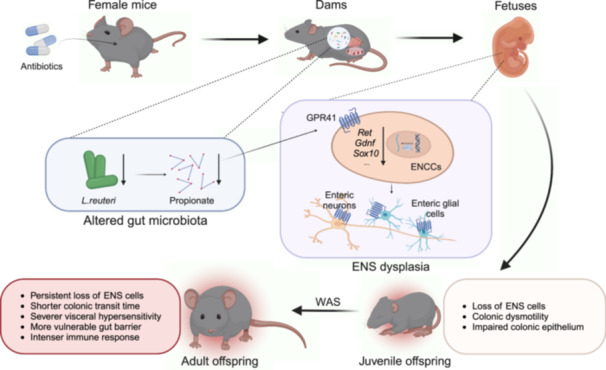

## INTRODUCTION

The maternal gut microbiota plays a crucial role in the health of offspring, particularly regarding the brain [1], immune system [2], and intestinal development [3]; however, the effects of the maternal gut microbiota on offspring during the preconception period remain underexplored. A recent study revealed that disruption of the gut microbiota during this critical period can predispose offspring to food allergies [4]. Notably, when the gut microbiota is disrupted, it can take up to 3–6 months to recover, often resulting in a significantly altered and less diverse microbial community [5]. Therefore, perturbations in the gut microbiota during the preconception period have the potential to persist throughout pregnancy, profoundly affecting the health of the mammalian offspring.

The enteric nervous system (ENS), composed of enteric neurons and enteric glial cells (EGCs), spans the entire gastrointestinal tract. Its ganglia, which interconnect neural fibers, form two principal plexuses: the myenteric plexus (MP) and submucosal plexus (SMP) [6]. The MP predominantly governs muscle contraction and relaxation, whereas the SMP regulates epithelial secretion and local blood flow [7]. It is widely recognized that the ENS influences gastrointestinal motility and secretion, encompassing immune modulation [8, 9], maintenance and repair of the epithelial barrier [10], and interaction with the central nervous system [11, 12]. In addition, it is involved in various pathogenic conditions, such as Hirschsprung's disease [13], gut–brain interaction disorders [14], inflammatory bowel disease [15], and colorectal cancer [16].

The ENS develops as enteric neuro crest cells (ENCCs) originating from the vagus nerve and sacral neural crest colonize the foregut. These cells proliferate and migrate throughout the entire intestine [17]. ENCCs differentiate into enteric neurons and EGCs to form the MP. Subsequently, the MP migrates inward to constitute the SMP and is distributed within the submucosal layer [18]. Notably, microorganisms can influence fetal ENS development in the uterus. Maternal *Chlamydia* infection has been associated with the loss of intestinal neurons and glial cells in the ENS of sheep fetuses [19]; however, research examining the impact of preconception maternal gut dysbiosis on fetal ENS development and disease susceptibility, particularly in relation to disorders of the gut–brain interaction, remains elusive.

In the present study, we created an antibiotic‐induced preconception maternal gut microbiota dysbiosis model to explore the characteristics of the ENS in offspring and their disease susceptibility (Figure S1). Our findings revealed that the use of antibiotic during the preconception period significantly affected the development and function of the ENS in offspring, increasing their susceptibility to water avoidance stress (WAS). Preconception antibiotic treatment altered the maternal gut microbiota and metabolic profile during pregnancy, particularly affecting the enrichment of *Limosilactobacillus reuteri* (*L. reuteri*) and cecal propionate levels. These changes downregulated the expression of ENS development‐related genes, including the suppression of *Gpr41* expression in the embryonic colon. Interestingly, supplementation with *L. reuteri* and propionate during gestation reversed these effects on ENS development in offspring. This study sheds light on the preconception risk factors for gut development and offers valuable insights into preconception healthcare.

## RESULTS

### Maternal preconception antibiotic exposure leads to ENS dysplasia in juvenile offspring

**Figure 1:**
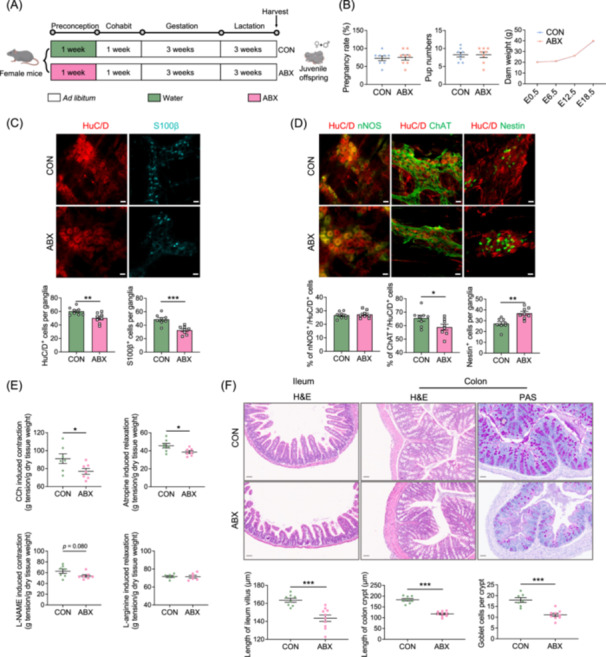
Maternal preconception antibiotics exposure leads to enteric nervous system (ENS) dysplasia in juvenile offspring. (A) Illustration of experimental model: Female mice were orally gavaged with antibiotics (ABX) or sterile water (CON) for 1 week, followed by discontinuation of antibiotics treatment and mating with male mice. After pregnancy and delivery, 3‐week‐old offspring (both male and female) were collected for analysis. (B) Pregnancy rate (*n* = 8–9), litter size (*n* = 8), and maternal weight during pregnancy (*n* = 8) per cage. (C) Immunostaining of HuC/D^+^ and S100^+^ cells in the myenteric plexus (MP) of the colon with the corresponding cell counts (*n* = 8), bar = 20 μm. (D) Immunostaining of ChAT^+^, nNOS^+^, and nestin^+^ cells in the MP of the colon with the corresponding cell counts (*n* = 8), bar = 20 μm. (E) Intervention with CCh, atropine, l‐NAME, and l‐arginine on colonic muscle strips of the offspring. The contraction or relaxation tension was measured, and the tension per gram of dry weight was calculated (*n* = 6–7). (F) Detection of villi in the ileum, colonic crypts, and goblet cell count in the colon of the offspring using hematoxylin and eosin (H&E) or periodic‐acid Schiff (PAS) staining (*n* = 8–10), bar = 50 μm. Mean ± SEM, *p < 0.05, **p < 0.01, ***p < 0.001 by unpaired Student's *t*‐test.

To explore how the preconception of maternal gut microbiota dysbiosis might affect ENS development in offspring, we administered antibiotics (ABX) to female mice. The mice were subjected to ABX treatment for 1 week, and the mice were mated with male mice in a 1:1 ratio during the subsequent week. Then, they were access to food and water *ad libitum* until the birth of their pups (Figure 1A). Compared with the control (CON) group, the ABX group exhibited no significant differences in pregnancy rate per cage, number of pups per dam, maternal weight during pregnancy, and weight of offspring, suggesting that the ABX regimen did not compromise dam fertility and nutritional status of offspring (Figure 1B, Figure S2A).

Lactation is pivotal for mammalian growth and development; therefore, we focused our analysis on the colonic MP of 3‐week‐old pups, including both sexes. Our observations revealed a noticeable difference in the proximal colonic MP of the ABX group, which appeared to be more dispersed with HuC/D^+^ staining in juvenile offspring (Figure 1C). We proceeded to count the HuC/D^+^ neurons, S100β^+^ EGCs, and two key neuronal subpopulations (neuronal nitric oxide synthase (nNOS)^+^ and choline acetyltransferase (ChAT)^+^) within each ganglion. Remarkably, we noted a reduction in HuC/D^+^ neurons and S100β^+^ EGCs in the colonic MP ganglia of juvenile mice from the ABX group (Figure 1C), along with a decrease in the proportion of ChAT^+^ neurons. However, the proportion of nNOS^+^ neurons was unaffected (Figure 1D). Notably, the colonic MP of ABX pups showed a marked increase in nestin^+^ cells, suggesting a heightened response to ENS damage (Figure 1D). It did not reveal any significant impact of sex on the results (Figure S2B).

Considering the developmental disturbances observed in colonic enteric neurons and their subtypes, we employed an ex vivo colonic muscle strip perfusion system to assess motor function impairments in ABX pups. Our findings showed that juvenile mice in the ABX group exhibited impaired cholinergic neurotransmission indicated by attenuated colonic muscle strip contractions in response to carbachol (*p* = 0.07) and diminished muscle strip relaxation upon the administration of atropine. l‐NAME and l‐arginine were used to assess nitric acid neurotransmission. The data demonstrated no notable differences in either l‐NAME‐induced contractions or l‐arginine‐induced relaxation between juvenile mice in the CON and ABX groups (Figure 1E). These results suggest a more pronounced impairment of the cholinergic pathway responsible for colonic contractions, potentially causing weakened colonic contractions and slowed transit. Likewise, sex was found to have no significant impact on the outcomes (Figure S2C).

Furthermore, we examined the effects of preconception ABX treatment on intestinal mucosal development in the 3‐week‐old offspring. Histological staining of the ileum and colon tissues showed that ABX pups had reduced villus length in the ileum, decreased crypt depth in the colon, and fewer goblet cells in the colonic crypt (Figure 1F). No significant disparities were noted among offspring of different sexes (Figure S2D). These findings indicate that preconception maternal ABX treatment not only led to aberrant ENS development and motor function in juvenile offspring but also impacted intestinal mucosal development.

### Offspring of dams treated with antibiotics exhibit persistent ENS dysplasia and increased disease susceptibility

**Figure 2:**
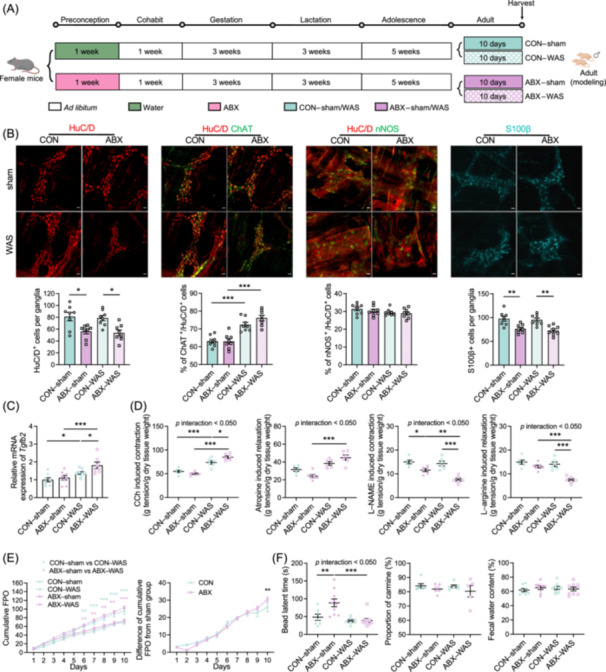
Adult offspring of dams treated with antibiotics during preconception exhibit persistent enteric nervous system (ENS) dysplasia and intestinal dysmotility, particularly when exposed to water avoidance stress (WAS). (A) Illustration of the experimental model: Female mice were orally gavaged with antibiotics (ABX) or sterile water (CON) for 1 week, followed by discontinuation of antibiotics treatment and mating with male mice. After pregnancy and delivery, 8‐week‐old adult male offspring underwent WAS or sham manipulation for 10 days. (B) Immunostaining of HuC/D^+^, ChAT^+^, nNOS^+^, and S100^+^ cells in the myenteric plexus (MP) of the colon in adult offspring, with the corresponding cell counts (*n* = 8), bar = 20 μm. (C) Relative mRNA expression of *Tgfb2* in the colon of adult offspring (*n* = 7–8). (D) Intervention with CCh, atropine, l‐NAME, and l‐arginine on colonic muscle strips of adult offspring. The contraction or relaxation tension was measured and the tension per gram of wet weight was measured (*n* = 6). (E) Sum of pellet counts during the modeling period and the difference between the WAS group and sham group (*n* = 6–10). (F) Evaluation of ENS‐mediated functions in adult offspring, including measurement of intestinal transit time by carmine red (*n* = 5–6), assessment of colonic transit rate by bead latency (*n* = 7–10), and determination of colonic secretory function by fecal water content (*n* = 8–9). Mean ± SEM, *p < 0.05, **p < 0.01, ***p < 0.001 by two‐way ANOVA followed by Tukey's (B, D, F) or Sidak's (C) multiple comparison test, and two‐way repeated measures ANOVA followed by Sidak's multiple comparison test (E).

To explore whether the developmental abnormalities of the ENS observed in juvenile offspring persisted into adulthood, we examined the colonic MP of adult offspring of dams treated with antibiotics during the preconception period. Given the known association between ENS abnormalities and gastrointestinal function disorders, we employed WAS to evaluate the susceptibility of these offspring to gut–brain interaction disorders (Figure 2A). To eliminate potential biases introduced by female sex hormones in the stress models, we limited the WAS study to male mice. We found that the number of HuC/D^+^ neurons and S100β^+^ EGCs remained reduced in the adult offspring of the ABX group, while the proportion of ChAT^+^ and nNOS^+^ neurons no longer showed significant differences. Exposure to WAS did not alter the counts of HuC/D^+^ neurons and S100β^+^ EGCs; however, it did induce an increase in the proportion of ChAT^+^ neurons in both the CON and ABX groups (Figure 2B). Furthermore, we found a significant increase in the mRNA expression of *Tgf‐β2* in the colon of adult offspring in the ABX–WAS group compared to the CON–WAS group (Figure 2C), suggesting a possible mechanism for the more severe ENS alterations observed. *Ex vivo* colon motor experiments revealed no significant differences in the cholinergic neural pathway; however, the NOergic neural pathway was impaired in the ABX offspring. Following WAS, the ABX offspring exhibited a more pronounced cholinergic response and a weaker NOergic response than the CON group (Figure 2D). These findings suggest that preconception ABX‐induced ENS dysplasia in the offspring may underlie the disrupted colon motility observed in response to WAS. Furthermore, colonic transit was slower in ABX offspring than in CON offspring, whereas the small intestine transit and fecal water content showed no significant differences (Figure 2E,F). Following WAS, both adult offspring displayed a reduction in colonic transit, with a greater effect observed in the ABX offspring (Figure 2E,F). These results confirm the presence of persistent colonic ENS abnormalities in the offspring of ABX dams, which contribute to weakened colonic contractions and a significant acceleration in response to stress.

The ENS is closely associated with gastrointestinal disorders. Based on our observations of ENS dysplasia and abnormal intestinal mucosa in the juvenile offspring of ABX dams, we further investigated visceral sensitivity, colonic barrier function, and colonic immunity in adult offspring. The application of WAS significantly increased colorectal distension–electromyography (CRD–EMG) activity in adult offspring, with a more pronounced response observed in the ABX group (Figures S3A,B, S4A,B). Offspring from the ABX group had shorter microvilli, wider tight junctions (TJs) between colonic epithelial cells, shorter TJ lengths, and a lower density of electron‐dense material (Figure S3C, Figure S4C,D). WAS resulted in the shortening of the microvilli and the widening of TJs in colonic epithelial cells. Notably, the ABX–WAS offspring displayed even shorter TJ lengths and decreased areas of electron‐dense material, along with indications of bridge granule fragmentation and microvillus deterioration (Figure S3C, Figure S4C,D). Further analysis revealed a significant downregulation of occludin mRNA expression in the ABX–sham group compared to the CON–sham group, confirming the presence of abnormal colonic barrier function in the adult offspring of ABX dams (Figure S3D). An impaired gut barrier serves as a crucial structural basis for inflammation. The ABX–WAS offspring exhibited significantly greater changes in mRNA expression of inflammatory markers, including *Tnf‐α* and *Csf‐1*, compared to the CON–WAS offspring (Figure S3E). Mast cells contribute to visceral hypersensitivity. Although the number of colonic mucosal mast cells was not significantly different between the CON and ABX offspring, the ABX offspring displayed a more pronounced response to WAS (Figure S3F). Furthermore, we analyzed the expression of inflammatory factors in the colons of the adult offspring; TNF‐α and IL‐17A presented significant differences between the ABX and CON groups (Figure S3G). These results are consistent with those of previous studies on irritable bowel syndrome (IBS) and its associated mechanisms [20, 21]. Based on these findings, we propose that the adult offspring of ABX dams exemplify the underdeveloped physiological foundations of the colonic ENS and colonic mucosal barrier. When exposed to WAS, they exhibit a striking ENS response, barrier disruption, and inflammation within the colon, ultimately leading to susceptibility and more severe abnormalities in intestinal motor, sensory, and barrier functions.

### ENS dysplasia emerges during embryonic development in the offspring of dams treated with antibiotics via the RET/GDNF/SOX10 signaling pathway

**Figure 3:**
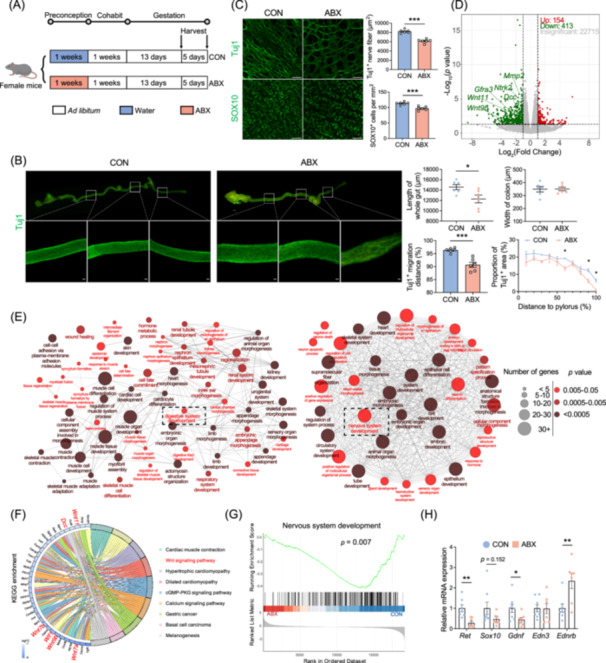
Enteric nervous system (ENS) dysplasia that emerges during the embryonic stage in the offspring of dams treated with antibiotics is mediated by the GDNF/RET/SOX10 signaling pathway. (A) Experimental model illustration: Female mice were orally gavaged with antibiotics (ABX) or sterile water (CON) for 1 week, followed by discontinuation of antibiotics and mating with male mice. After pregnancy, embryos at E13.5 and E18.5 were collected for examination, irrespective of sex. (B) Tuj1 staining of the entire intestine of E13.5 fetal mice. Measurements included the length of the whole intestine, width of the colon, and migration distance of Tuj1^+^ cells. The intestine was divided into 10 equal parts, and the proportion of Tuj1^+^ staining in each segment was calculated (*n* = 6, scale bar = 50 μm). (C) Tuj1 and Sox10 staining of the colon in E18.5 fetal mice. The area occupied by Tuj1^+^ neurons and the number of Sox10^+^ cells per square millimeter were quantified (*n* = 6, scale bar = 50 μm). (D) Volcano plot displays the upregulation or downregulation of gene expression in the fetal mouse colon in the ABX group (*n* = 3). (E) Enrichment analysis of embryonic colon gene expression for gene ontology (GO). Nodes were colored based on *p*‐values, and node size corresponded to the number of genes. Edges represented statistically significant associations between GO terms. (F) Kyoto Encyclopedia of Genes and Genomes (KEGG) enrichment analysis for embryonic colon gene expression selecting the top nine pathways based on the lowest adjusted *p*‐value. (G) Gene set enrichment analysis (GSEA) demonstrates the downregulation of “Nervous system development” in the ABX group. The Wilcoxon test, followed by FDR correction, was used. (H) Relative mRNA expression of key genes involved in ENS development in the colon of fetal mice (*n* = 7–8). Mean ± SEM, *p < 0.05, **p < 0.01, ***p < 0.001 by unpaired Student's *t*‐test or two‐way ANOVA followed by Tukey's multiple comparison test (B, C, H).

Given the profound and enduring developmental abnormalities observed in the ENS and intestinal mucosa of the offspring of ABX dams, we postulated that these aberrations might have originated during the embryonic stage, which is a crucial period for growth and development. Therefore, we procured intestinal tissues from embryonic day (E) 13.5 and E18.5 from the CON and ABX groups to assess the migration and proliferation of the ENS (Figure 3A). E13.5 signifies the time point at which the ENS migrates from the foregut to the anus, while E18.5 marks the final day of embryonic development for the ENS. As expected, the E13.5 embryos from the ABX group exhibited a notably shorter intestine length from the pylorus to the anus, although the colon width remained unaffected (Figure 3B). Additionally, the migration distance percentage of Tuj1^+^ nerve fibers in the embryos of the E13.5 embryos from the ABX group was significantly lower. When the intestine was further segmented into 10 equal parts, a discernible decrease in the density of Tuj1^+^ staining was observed in the ABX group, which was particularly significant in the colon (Figure 3B). Subsequently, Tuj1^+^ and SOX10^+^ staining was performed on the colon tissue at E18.5, revealing a substantial reduction in the Tuj1^+^ staining area and SOX10^+^ cell count in the ABX group (Figure 3C), indicating the compromised proliferation efficiency of the ENS.

To delve deeper into the potential abnormalities in gene expression and the underlying mechanisms associated with colonic ENS development in embryos of the ABX group, we conducted a transcriptome analysis of colon tissues derived from E18.5. Our findings revealed 567 differentially expressed genes in the ABX group, of which 154 were upregulated, and 413 were downregulated (Figure 3D, Figure S5A). Several genes known for their close association with ENS development, such as *Ntrk2*, *Gfra3*, and *Dcc*, were significantly downregulated in the ABX group (Figure 3D). The Gene Ontology (GO) enrichment analysis revealed that the upregulated genes were linked to developmental processes in the ABX group (Figure S5B). Interestingly, both “Digestive system development” and “Nervous system development” were among the enriched pathways (Figure 3E). Kyoto Encyclopedia of Genes and Genomes (KEGG) pathway enrichment analysis highlighted the significant enrichment of the “Wnt signaling pathway” (Figure 3F), a crucial signaling pathway for hindgut development, corroborating the observed colon developmental abnormalities in the ABX offspring. Further gene set enrichment analysis (GSEA) demonstrated significant downregulation of multiple pathways enriched in both the GO and KEGG databases, encompassing “Nervous system development,” “Muscle tissue development,” and the “Wnt signaling pathway” in the ABX embryos (Figure 3G, Figure S5C–F).

The proliferation, migration, and differentiation of the ENS predominantly involve the RET/GDNF and EDN3/EDNRB signaling pathways, with SOX10 playing a pivotal role as a transcription factor in both. Analysis of the mRNA expression of these key molecules in the E18.5 embryonic colon revealed the downregulation of *Ret, Gdnf*, and *Sox10* in the ABX group (Figure 3H). Interestingly, the mRNA expression of *Ednrb* was significantly upregulated in the colons of ABX fetuses (Figure 3H), suggesting a compensatory adaptation to counteract the downregulation of Ret/Gdnf signaling. These observations suggest that preconception ABX treatment can disrupt normal embryonic colon ENS development, primarily through signaling pathways involving RET/GDNF/SOX10.

### Preconception antibiotic exposure profoundly influences the maternal gut microbiome and metabolome

**Figure 4:**
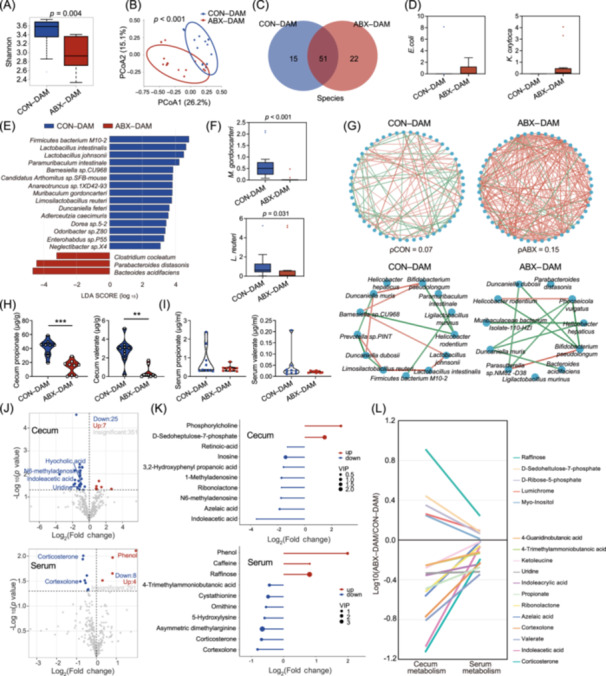
Preconception antibiotics treatment shapes the maternal gut microbiome and metabolome throughout gestation. (A) The Shannon index of maternal gut microbiota at the species level at E18.5 was computed. Variations were assessed using the Wilcoxon test with FDR correction (*n* = 12–13). (B) Principal coordinate analysis (PCoA) analysis of maternal gut microbiota using Bray–Curtis distances at the species level. Differences were determined using the PERMANOVA test. (C) Venn diagram revealing shared species and differential species between the two groups. (D) Relative abundances of *Escherichia coli* and *Klebsiella oxytoca*. (E) Linear discriminant analysis effect size (LEfSe) analysis of 51 shared species to identify significant abundance differences between groups. The Wilcoxon test with FDR correction was used to calculate differences. The Linear discriminant analysis (LDA) score was computed to estimate the impact of species abundance on differential effects. (F) Relative abundances of *Muribaculum gordoncarteri* and *Limosilactobacillus reuteri*. (G) Co‐occurrence network of maternal gut microbiota. The top two graphs were constructed using 51 shared species, and the bottom two graphs selected dominant species with an abundance >1%. Edges indicated statistically significant associations between species, with red showing positive and green showing negative correlations. Spearman's rank correlation test was used for the correlation analysis. (H) Concentrations of propionate and valerate in the cecum of maternal mice (*n* = 9). (I) Concentrations of propionate and valerate in the serum of maternal mice (*n* = 9). (J) Volcano plot illustrating the upregulation or downregulation of metabolites in the cecum and serum of maternal mice (*n* = 9). (K) Bar graphs displaying the top 10 differential metabolites with the largest fold changes in the cecum and serum, where the size of the circles corresponded to the VIP value. (L) Calculation of the ratio of metabolites in the cecum and serum of maternal mice (ABX–DAM/CON–DAM). Trends on the same side of the *x*‐axis indicated similar changes in the cecum and serum. Significance levels: *p < 0.05, **p < 0.01, ***p < 0.001 indicated by Mann–Whitney *U* test (H, I). CON, control; ABX, antibiotics.

To delineate the mechanisms leading to abnormal ENS development in fetal mice in the ABX group, we conducted a comprehensive assessment of the maternal gut microbiota and its metabolites. Fecal samples were collected from dams at E18.5 for metagenomic analysis. Our findings revealed a notable decrease in alpha diversity in the microbiota of preconception ABX dams, as indicated by the Shannon and Simpson indices (ABX–DAM), compared to that in the control group (CON–DAM) (Figure 4A, Figure S6A). Principal coordinate analysis (PCoA) based on the Bray–Curtis distance further confirmed the distinct clustering between the ABX–DAM and CON–DAM groups (Figure 4B, Figure S6B). Specifically, at the phylum level, significant differences were observed in the abundances of Firmicutes and Bacteroidetes between the two groups, resulting in a significantly decreased F/B ratio in the ABX–DAM group (Figure S6C,D). Linear discriminant analysis effect size (LEfSe) analysis identified *Lactobacillus* and *Paramuribaculum* as key genera distinguishing the CON–DAM group, whereas *Bacteroides* and *Parabacteroides* had the most significant impact on the ABX–DAM group (Figure S6E–G). The ABX–DAM group harbored a greater number of unique species, including potentially harmful pathogens, such as *Escherichia coli* and *Klebsiella oxytoca* (Figure 4C,D). Among the common species, many beneficial bacteria were depleted in the ABX–DAM group, including *Lactobacillus intestinalis*, *Limosilactobacillus reuteri* (*L. reuteri*), and *Muribaculum gordoncarteri* (*M. gordoncarteri*) (Figure 4E,F).

To gain deeper insight into the interrelationships within the maternal gut microbiota, we employed a co‐occurrence network analysis. Despite the decreased alpha diversity in the fecal samples from the ABX–DAM group, their internal microbial interactions were more complex. Within the predominant species, interactions in the ABX–DAM group remained intricate and were dominated by negative correlations (Figure 4G). This underscores the competitive relationships and nuanced regulatory mechanisms involved in microbial reconstitution in the ABX–DAM group. Moreover, our analysis revealed the depletion of functional pathways involved in amino acid and nucleotide synthesis in the ABX–DAM group, whereas those pentose phosphate pathways were enriched (Figure S7A–D). These findings suggest that preconception ABX treatment significantly alters the maternal gut microbiome and metabolome, potentially contributing to the observed abnormalities in ENS development in the offspring.

Due to variations in the abundance of bacteria capable of producing short‐chain fatty acids (SCFAs) between the CON–DAM and ABX–DAM groups, we evaluated SCFA levels in the cecal contents and serum of maternal mice at E18.5. In the ABX–DAM group, the concentrations of acetate, propionate, butyrate, and valerate in the cecal contents were notably lower than those in the CON–DAM group, with propionate and valerate showing particularly large fold changes. However, no appreciable differences were observed in the serum levels of these SCFAs (Figure 4H,I; Figure S8A,B). To investigate whether there were metabolite differences beyond SCFAs, we employed a targeted metabolome approach (Figure S8C, Table S3) to further analyze the cecal contents and serum of maternal mice at E18.5, which revealed a distinct separation between the groups based on orthogonal partial least squares discriminant analysis (OPLS‐DA) (Figure S8D). Using volcano and bar plots, we identified key metabolites in the cecal contents, including indoleacetic acid and N6‐methyladenosine, both of which were consistently downregulated in the ABX–DAM group and strongly correlated with each other (Figure 4J,K, Figure S8E). Additionally, KEGG pathway enrichment analysis demonstrated distinct metabolic capabilities between the two groups (Figure S8F). Analysis of the serum metabolome also showed a clear separation between the groups, with tighter intragroup clustering compared to that of the cecal contents (Figure S8G). Volcano and bar plots highlighted corticosterone and cortexolone as the two compounds with the largest downregulated fold‐changes in the ABX–DAM group, exhibiting a robust correlation (Figure 4L,M, Figure S8H). Phenol and caffeine concentrations were markedly upregulated in the ABX–DAM group. KEGG pathway enrichment analysis indicated several significantly downregulated metabolic pathways in the ABX–DAM group (Figure S8I). Although there was minimal overlap in the differential metabolites between the cecal contents and serum, the trends in concentration changes between the two were consistent (Figure 4L). Collectively, our findings highlight the substantial modifications in the gut microbiota and its metabolites in the ABX group, which may contribute to the emergence of ENS abnormalities.

### Multi‐omics analysis revealed that *L. reuteri*, *M. gordoncarteri*, and propionate significantly influence ENS development

**Figure 5:**
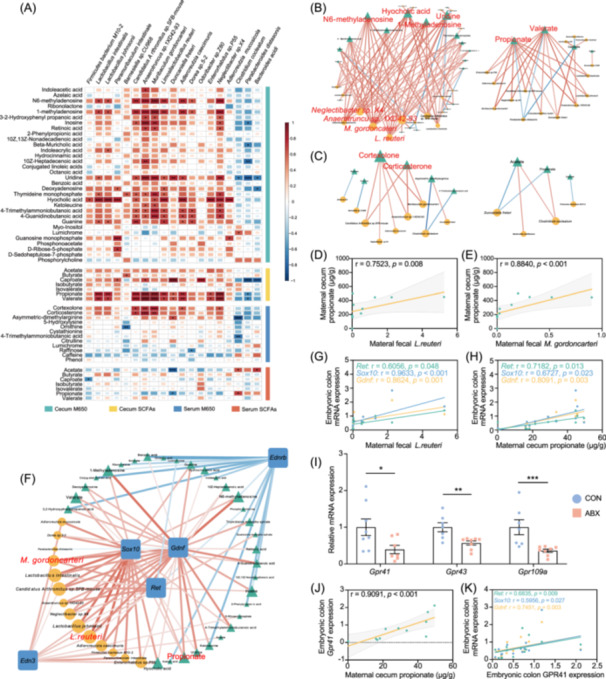
Multi‐omics analysis showed that *L. reuteri*, *M. gordoncarteri*, and propionate impacted enteric nervous system (ENS) development. (A) Heatmap showing the correlation between differentially abundant fecal species and differentially abundant cecal and serum metabolites in E18.5 maternal mice (*n* = 11). The larger the square and the darker the color, the larger the |*r*| value. *p < 0.05, **p < 0.01, ***p < 0.005. (B) Interaction network between differentially abundant species and differentially abundant cecal metabolites in maternal mice. Triangular nodes represent metabolites, while circular nodes represent bacterial species. The size of the nodes indicates the degree of the network. Red edges represent positive correlations; blue edges represent negative correlations. (C) Similar to (B), an interaction network was created between differentially abundant species and differentially abundant serum metabolites in maternal mice. (D, E) Scatter plots and linear regression analysis show the correlation between the abundance of *L. reuteri* and *M. gordoncarteri* in maternal feces and the concentration of propionate in the cecum of maternal mice. (F) Similar to (B), an interaction network was created between differentially abundant species, differentially abundant cecal metabolites, and mRNA expression related to ENS development in maternal mice (*n* = 11). Square nodes represent mRNA expression. (G, H) Scatter plots and linear regression analysis showing the correlation between the abundance of *L. reuteri* in maternal feces and the concentration of propionate in the cecum, and the mRNA expression of *Ret, Gdnf*, and *Sox10* in the embryonic gut. (I) Relative mRNA expression of short‐chain fatty acid (SCFA) receptors in the embryonic intestine. (J) Scatter plot and linear regression analysis showing the correlation between cecal propionate concentration and embryonic *Gpr41* mRNA expression in maternal mice. (K) Scatter plots and linear regression analysis showing the correlation between embryonic *Gpr41* mRNA expression and the mRNA expression of *Ret, Gdnf*, and *Sox10* in the embryonic gut. Correlation analysis was performed using the Spearman's rank correlation test. Mean ± SEM, *p < 0.05, **p < 0.01, ***p < 0.001 by the unpaired Student's *t*‐test.

Using a multi‐omics approach, we investigated the key microbial species and metabolites that are associated with the development of the ENS. Initially, we employed Spearman's rank correlation test to identify components with high connectivity and top rankings within the interaction network between species and metabolites, considering these to be of significant importance due to their central position and close interactions. We designate these as core differential species and metabolites with tightly associated relationships. Specifically, *M. gordoncarteri*, *L. reuteri*, *Neglectibacter sp. X4*, and *Anaerotruncus sp. 1XD42‐93* were identified as core species (Figure 5A,B). Additionally, hyocholic acid, uridine, N6‐methyladenosine, 1‐methyladenosine, propionate, and valerate in the cecal contents, as well as corticosterone and cortexolone in the serum, were identified as core metabolites (Figure 5C).

Scatter plots illustrate the relationship between propionate, *M. gordoncarteri* and *L. reuteri* (Figure 5D,E). Furthermore, our analysis uncovered PWY‐6527, PWY6317, 1CMET2‐PWY, and PWY7977 as the core metabolic pathways (Figure S9A–E). Notably, *L. reuteri* and other *Lactobacillus* species significantly contributed to the depleted pathways in the antibiotic‐treated dams, whereas opportunistic pathogens, such as *Escherichia coli*, contributed more to the enriched pathways, highlighting the distinct microbial profiles between the two groups (Figure S9A–E).

To identify the crucial metabolites and core bacterial species influencing embryonic ENS development, we correlated the differentially abundant maternal bacteria, cecal metabolites, and mRNA expression levels of five crucial ENS developmental genes in the embryonic colon (previously examined in Figure 3H). The RET/GDNF and SOX10 pathways exhibited stronger correlations than the EDN3/EDNRB pathway (Figure 5F). Significant positive correlations were observed between *M. gordoncarteri*, *L. reuteri*, propionate, valerate, with *Ret, Gdnf, Sox10*, and *Edn3* expression (Figure 5F). Given the lower concentration of valerate than propionate, we identified propionate in the cecal content as a key metabolite. Additionally, serum corticosterone and cortexolone levels were positively correlated with the mRNA expression of *Ret* and *Gdnf* in the embryonic colon (Figure S10A). Analysis of metabolite receptors in the fetal colon revealed significant downregulation or decreasing trends in the expression of *Tgr5, Tlr2, Nr3c1*, and *Nr3c2* in the ABX group, with the exception of *Tlr4* (Figure 5I, Figure S10B). Exploring the mechanisms by which maternal metabolites exert their effects, we found a consistent positive correlation between metabolite levels and the mRNA expression of corresponding receptors in the embryonic colon. Propionate and valerate showed the strongest correlation with *Gpr41* (Figure 5J and Figure S10C). The relationship between *Gpr41* expression and *Ret, Gdnf*, and *Sox10* expression was particularly pronounced (Figure 5K, Figure S10D), suggesting the critical role of GPR41 in embryonic ENS development. Based on these findings, we propose that the core bacterial species *M. gordoncarteri* and *L. reuteri* influence propionate production and facilitate ENS development via GPR41, which is impeded by ABX treatment.

### *L. reuteri* plays a crucial role in ENS development via the production of propionate

**Figure 6:**
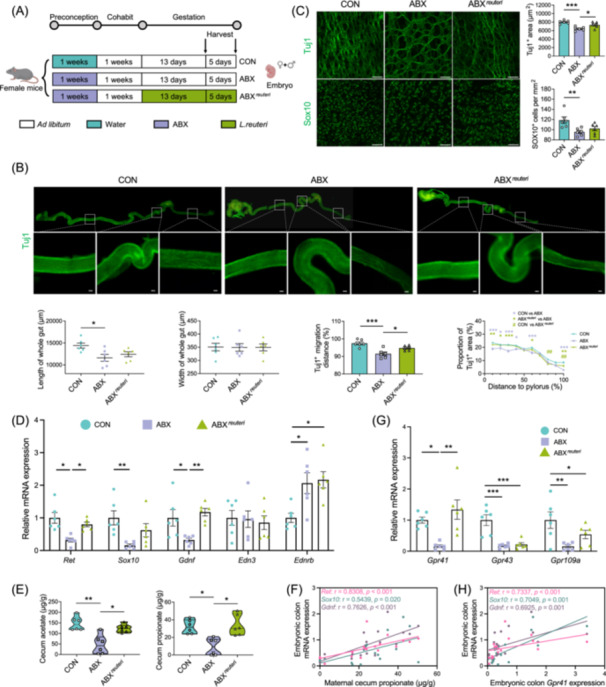
*Limosilactobacillus reuteri* plays a crucial role in enteric nervous system (ENS) development during gestation. (A) Experimental model illustration: Female mice were orally administered antibiotics (ABX) or sterile water (CON) for 1 week, followed by discontinuation of antibiotics use and mating with male mice. Subsequently, some pregnant mice previously exposed to ABX were subjected to *L. reuteri* intervention during pregnancy (ABX^*reuteri*^). Embryos at E13.5 and E18.5 were collected for examination, without regard to sex. (B) Tuj1 staining of the entire intestine of E13.5 fetal mice. Measurements included the length of the entire intestine, width of the colon, and migration distance of Tuj1^+^ cells. The intestine was divided into 10 equal parts, and the proportion of Tuj1^+^ staining in each segment was calculated (*n* = 6, scale bar = 50 μm). (C) Tuj1 and Sox10 staining of the colon in E18.5 fetal mice. The area occupied by Tuj1^+^ neurons and the number of Sox10^+^ cells per mm^2^ were quantified (*n* = 6, scale bar = 50 μm). (D) Relative mRNA expression of key genes involved in ENS development in the fetal mouse colon (*n* = 6). (E) Concentrations of acetate and propionate in the cecum of maternal mice (*n* = 6). (F) Scatter plot showing the correlation between acetate and propionate concentrations in the maternal cecum and mRNA expression related to ENS development in the fetal colon. (G) Relative mRNA expression of short‐chain fatty acid (SCFA) receptors in fetal intestines. (H) Scatter plot and linear regression of the correlation between fetal *Gpr41* mRNA expression and fetal intestinal *Ret, Gdnf*, and *Sox10* mRNA expression. Correlation analysis was performed using the Spearman's rank correlation test. Mean ± SEM, *p < 0.05, **p < 0.01, ***p < 0.001 by one‐way ANOVA followed by Tukey's multiple comparison test (B–D, G) or Kruskal–Wallis test followed by Dunn's multiple comparison test (E).

Considering that *M. gordoncarteri* is exclusively observed in rodents and may exhibit pathogenicity, we focused on *L. reuteri* because of its beneficial metabolites, such as SCFAs and tryptophan derivatives, and its profound effect on microbial community structure. To identify probiotics that are beneficial for ENS development and have potential clinical applications, we chose *L. reuteri* as the intervention throughout gestation. This was performed to mitigate any adverse effects of preconception ABX treatment on embryonic ENS development as early as possible (Figure 6A). Preconception ABX treatment influenced colon length, and the Tuj1^+^ fiber migration percentage and density at E13.5. However, gestational supplementation with *L. reuteri* had a remarkable ameliorative effect on ENS migration and density (Figure 6B). Subsequent staining of E18.5‐mouse embryo colons for Tuj1^+^ fibers and Sox10^+^ cells revealed that *L. reuteri* effectively rescued the Tuj1^+^ staining area affected by preconception ABX treatment, although its impact on Sox10^+^ cell counts was less pronounced (Figure 6C). mRNA expression analysis of embryonic colons demonstrated that *L. reuteri* supplementation restored the downregulation of the RET/GDNF pathway induced by ABX preconception treatment (Figure 6D).

We analyzed SCFA levels in the maternal cecal contents and serum during the late gestational period. Significantly higher levels of acetate and propionate were observed in the ABX^*reuteri*^ group, whereas butyrate and valerate levels exhibited less significant changes (Figure 6F, Figure S11A). Serum levels of SCFAs showed an upward trend (Figure S11B). In vitro cultivation experiments have also demonstrated that *L. reuteri* is capable of producing acetate and propionate (Figure S11C). Correlation analysis of the mRNA expression of *Ret, Gdnf*, and *Sox10* revealed significant correlations with propionate and valerate levels, surpassing those with acetate and butyrate levels (Figure 6G, Figure S11D). Notably, embryonic colon *Gpr41* mRNA expression showed a strong correlation with maternal cecum propionate and demonstrated a significant recovery in the ABX^*reuteri*^ group (Figure 6H, Figure S11E). Additionally, treatment with *L. reuteri* rescued the expression of *Tgr5* (Figure S11F). Furthermore, embryonic colon *Gpr41* exhibited significant positive correlations with *Ret, Gdnf*, and *Sox10* mRNA expression, emphasizing the vital role of propionate in embryonic ENS development (Figure S11G).

**Figure 7:**
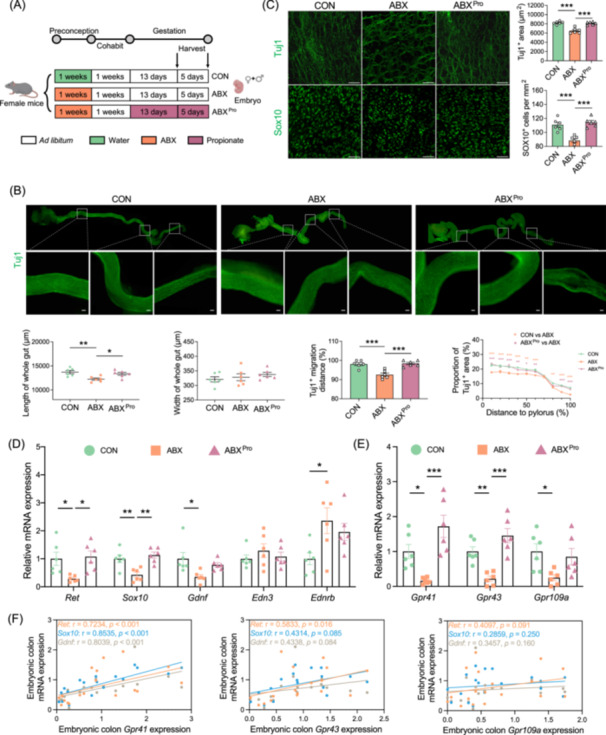
Propionate may promote enteric nervous system (ENS) development through the GPR41–GDNF/RET/SOX10 signaling pathway. (A) Experimental model illustration: Female mice were orally administered antibiotics (ABX) or sterile water (CON) for 1 week, followed by discontinuation of antibiotics and mating with male mice. Subsequently, some pregnant mice previously exposed to ABX were subjected to propionate intervention during pregnancy (ABX^Pro^). Embryos at E13.5 and E18.5 were collected for examination, regardless of sex. (B) Tuj1 staining of the entire intestine of E13.5 fetal mice. Measurements included the length of the entire intestine, width of the colon, and migration distance of Tuj1^+^ cells. The intestine was divided into 10 equal parts, and the proportion of Tuj1^+^ staining in each segment was calculated (*n* = 6, scale bar = 50 μm). (C) Tuj1 and Sox10 staining of the colon in E18.5 fetal mice was performed, and the area occupied by Tuj1^+^ neurons and the number of Sox10^+^ cells per mm^2^ were quantified (*n* = 6, scale bar = 50 μm). (D) Relative mRNA expression of key genes involved in ENS development in the fetal mouse colon following intervention with propionate (*n* = 6). (E) Relative mRNA expression of short‐chain fatty acid (SCFA) receptors, including *Gpr41, Gpr43*, and *Gpr109A* in the fetal colon (*n* = 6). (F) Scatter plots and linear regression analysis show the correlation between the mRNA expression of SCFAs receptor and mRNA expression of *Ret, Gdnf*, and *Sox10* in the embryonic colon (*n* = 18). *p < 0.05, **p < 0.01, ***p < 0.005. Correlation analysis was performed using the Spearman's rank correlation test (F). Mean ± SEM, analyzed by one‐way ANOVA followed by Tukey's multiple comparison test (A–E).

To further validate the crucial role of propionate in ENS development, we conducted gestational intervention with propionate in maternal mice (Figure 7A). After the administration of sodium propionate, a significant increase in propionate levels was observed in the cecum of the dams, which was 16.45 times higher than in dams supplemented with *L. reuteri* (Figure S12A). We observed that supplementation with propionate restored colon shortening as well as the migration and proliferation of the ENS, which were affected by preconception antibiotic exposure at E13.5 (Figure 7B). Subsequent staining of Tuj1^+^ fibers and Sox10^+^ cells in the colon at E18.5 revealed that propionate significantly rescued the Tuj1^+^ staining area and that Sox10^+^ cell counts were affected by preconception antibiotic exposure (Figure 7C). The mRNA expression analysis of embryonic colons showed that propionate supplementation restored the ABX‐induced downregulation of *Ret, Gdnf*, and *Sox10* (Figure 7D). Preconception antibiotic exposure led to the downregulation of the mRNA expression levels of SCFA receptors in maternal mice, and propionate intervention restored the expression of *Gpr41* and *Gpr43* (Figure 7E). Furthermore, the mRNA levels of *Gpr41* exhibited a significant positive correlation with the mRNA expression of *Ret, Gdnf*, and *Sox10*, suggesting that propionate regulates the expression of ENS development‐related genes through the GPR41 pathway (Figure 7F). In contrast, valerate had a relatively weak effect on ENS development (Figure S12B–E).

Collectively, our findings suggest that supplementation with *L. reuteri* after ABX treatment during pregnancy can increase cecal propionate levels. This modulated the expression of *Ret*, *Gdnf*, and *Sox10* through the GPR41 signaling pathway, promoting the amelioration of ENS dysplasia induced by preconception ABX treatment.

## DISCUSSION

The ENS is a vital component of the digestive tract and plays a pivotal role in various gastrointestinal conditions. Our findings revealed that the preconception mof aternal antibiotic exposure significantly alters ENS development in both juvenile and adult offspring, predisposing them to conditions such as central stress in adulthood. Remarkably, these alterations occurred during the embryonic stage. This study indicates that preconception maternal antibiotic exposure impacts both the gut microbiota composition and metabolic signatures in the cecal contents and serum. Specifically, antibiotics block the production of propionate by *L. reuteri* during pregnancy, may disrupt the GPR41–RET/GDNF/SOX10 signaling pathway, and ultimately impede embryonic ENS maturation.

Previous studies have established that the maternal gut microbiota influences the development of the digestive, nervous, and immune systems in the offspring. For instance, prenatal exposure to food additives or organic pollutants can affect colonic crypt development in the offspring by altering the maternal gut microbiota [3, 22]. Moreover, gut dysbiosis during the preconception period has been linked to immune dysregulation in the offspring, although the exact mechanisms remain incompletely understood [4]. Prenatal ENS development is affected by various maternal factors. Limited protein intake and vitamin A deficiency during gestation can affect ENS function in the offspring [23, 24]. In our study, juvenile mice from dams exposed to antibiotics exhibited reduced enteric neuron, and EGCs counts per ganglion accompanied by sluggish colonic motility. This is the first study to demonstrate that imbalances in the gut microbiota of mothers before conception can affect ENS development in their offspring.

Visceral hypersensitivity is a crucial pathophysiological mechanism in IBS. Mucosal barrier function and inflammation are associated with visceral hypersensitivity [25, 26], and recent studies implicate the involvement of enteric neurons and EGCs [27, 28]. Our results showed that adult offspring from the antibiotic‐treated group exhibited increased visceral sensitivity, higher susceptibility to WAS, abnormal ENS, compromised colonic mucosal barrier function, and elevated inflammatory cytokine levels. These findings offer insights into increased visceral sensitivity to WAS, shedding light on the intricate relationship between maternal gut microbiota and offspring ENS development and suggesting that the preconception of maternal gut dysbiosis is highly likely to be a risk factor for dysplasia in human offspring.

The embryonic phase holds utmost significance in the development of the ENS. Our data showed a downregulation in the mRNA expression of *Ret, Gdnf*, and *Sox10* in the colon of embryos from dams treated with antibiotics. This suggests that the abnormal ENS development in embryos from ABX dams is closely linked to the GDNF/RET/SOX10 pathway. Interestingly, the mRNA expression of *Ednrb* was upregulated, possibly as a compensatory mechanism. Moreover, blocking the Wnt pathway disrupts the normal proliferation and differentiation of colonic epithelial tissues [29]. Our study found that the Wnt pathway was downregulated in the offspring of the ABX group, providing an explanation for the intestinal abnormalities and mucosal barrier dysfunction observed in these mice. To our knowledge, this is the first study to perform transcriptome analysis of the embryonic colon in response to maternal gut microbiota dysbiosis in mice, thereby advancing our understanding of how the maternal microbiota affects embryonic ENS development.

Our results suggest that the gut microbiota of ABX dams reduces the number of potentially beneficial neutral bacteria during pregnancy. Among these bacteria, *L. reuteri* stands out because of its ability to produce various beneficial metabolites vital for bodily functions and its profound impact on microbial community structure [30, 31]. Previous studies have shown that *L. reuteri* can effectively enhance gut and central nervous system development in offspring whose mothers are exposed to a high‐fat diet, lipopolysaccharides, or antibiotic [32, 33]. In our multi‐omics analysis, *L. reuteri* emerged as a key species contributing to ENS development. Furthermore, our interventional studies with *L. reuteri* during pregnancy in ABX dams showed that *L. reuteri* partially improved ENS density and migration, further validating its importance in ENS development and paving the way for potential clinical probiotic treatment strategies. We also observed that the microbial interactions within the ABX group were more complex. One possible explanation for this is that following the antibiotic‐induced disruption of the maternal gut microbiota, bacteria‐carrying resistance genes were selectively enriched [34]. Studies have indicated that after antibiotic exposure, bacteria with resistance genes are more likely to colonize but are less likely to survive [35], resulting in a diverse yet mutually inhibitory microbial community. Furthermore, antibiotic exposure promotes the colonization and survival of beneficial genera such as *Bacteroides* and *Bifidobacterium*. This phenomenon can be attributed to the fact that the genomes of these taxa are rich in genes involved in the degradation of dietary polysaccharides and mucin proteins, which may provide metabolic advantages that facilitate community recovery [36]. Notably, an increased abundance of *Escherichia coli* has been associated with abnormal central nervous system development in offspring due to factors such as maternal stress and a high‐fat diet [37, 38]. *Klebsiella oxytoca*, another opportunistic pathogen, has been linked to the onset and poor prognosis of several diseases [39]. These findings underscore the detrimental effects of preconception antibiotic treatment on maternal gut microbiota, which can have far‐reaching consequences that impede offspring development.

Analysis of the cecal contents revealed significantly reduced levels of SCFAs in ABX dams. The SCFAs in the serum of ABX dams also exhibited a similar trend, albeit the difference was not statistically significant. We propose that metabolites are primarily produced through bacterial fermentation in the feces, thus exhibiting more pronounced concentration variances. Once these metabolites are absorbed into the bloodstream, they are utilized by the body, and the blood has self‐regulating mechanisms that may obscure significant differences in serum metabolite levels. Another study has also reported a comparable finding [39]. Previous studies have documented the profound effects of acetate, propionate, and butyrate in promoting ENCC proliferation and migration both in vitro and in vivo [40, 41]. Among SCFAs, propionate is particularly significant, as it functions during the embryonic stage, driving the development of the pancreatic autonomic nervous system via GPR41 activation [42]. In our comprehensive multi‐omics analysis, propionate emerged as a critical regulator of the genes involved in ENS development. Further investigation established a robust correlation between embryonic colon *Gpr41* expression and maternal cecum propionate levels, suggesting that propionate exerts its developmental influence on the ENS through the GPR41 signaling pathway. Consistent with previous reports [43, 44], our study demonstrated that *L. reuteri* enhances host propionate production. Intervention with *L. reuteri* and propionate in ABX dams further confirmed the pivotal role of the GPR41–GDNF/RET/SOX10 pathway in ENS development.

However, it is worth noting that the preconception gut microbiota not only shapes the maternal gut microbiota during pregnancy but also has the potential to impact the activity and epigenetic modifications of reproductive cells [45, 46], which were challenging to exclude in this study. More in‐depth research is warranted to elucidate the molecular mechanisms by which GPR41 mediates ENS development‐related gene expression.

## CONCLUSION

The findings of this study provide compelling evidence for the significance of the preconception of gut microbiota in health. This bridges a crucial gap in our understanding of the relationship between the preconception maternal gut microbiota, ENS development, and disease susceptibility of offspring. Furthermore, we identified key species and metabolites that foster ENS development during embryogenesis, paving the way for future research and potential interventions.

## METHODS

### Animals

Female (7 weeks old) and male (9 weeks old) C57BL/6J mice were used from the Department of Laboratory Animal Science, Peking University Health Science Center, Beijing, China, and reared as specific pathogen‐free (SPF) mice. The animals were housed in SPF facilities with a 12‐h light–dark cycle (lights on from 6:00 AM to 6:00 PM). Upon entering the SPF facility, all animals underwent a 1‐week acclimation period before proceeding to the next stage of the experiment. Unless otherwise specified, all the mice had *ad libitum* access to sterilized food (Xietong Shengwu) and water.

For the experimental investigation of the impact of gut microbiota dysbiosis on the development of ENS in juvenile and adult offspring, the ABX group of female mice received a daily oral gavage of a mixture containing neomycin (Innochem) (100 mg/kg), metronidazole (Aladdin) (100 mg/kg), and vancomycin (Innochem) (50 mg/kg) in the morning and evening, along with *ad libitum* access to ampicillin (Aladdin) (1 mg/mL) in the drinking water, to simulate the preconception gut microbiota dysbiosis state. The CON group received an oral gavage of the vehicle (sterile water). Oral gavage was continued for 1 week, and the mice were mated with male mice in a 1:1 ratio during the subsequent week. The appearance of the vaginal plug was designated as E0.5. After the vaginal plug was observed, the pregnant mice were transferred to new SPF cages and provided *ad libitum* access to food and water. Body weight was measured weekly. After parturition, the offspring were raised on SPF bedding, and their body weights were recorded weekly with *ad libitum* access to food and water. A subset of the offspring (unspecified sex) was killed at 3 weeks of age for tissue collection. Considering the influence of hormonal fluctuations on visceral sensitivity, subsequent WAS modeling, testing, and tissue collection were performed only on non‐euthanized male mice. Each offspring was considered as one sample size.

To experimentally investigate the impact of gut microbiota dysbiosis on embryonic ENS development, female mice in the ABX and CON groups underwent ABX or vehicle treatment and were mated accordingly. Fetal mice were collected at E13.5 and E18.5. Additionally, serum, cecal content, and fecal samples were obtained from the dams. Data from all fetuses (unspecified for sex) derived from a single dam were averaged to represent one sample size.

For experimental intervention with *L. reuteri* in the context of gut microbiota dysbiosis, treatment procedures for the ABX and CON groups remained unchanged. In the ABX^*reuteri*^ group, female mice underwent the aforementioned ABX treatment, and starting from E0.5, they received daily oral gavage of 200 μL of *L. reuteri* (BNCC) at a concentration of 1 × 10^9^ CFU/mL until the completion of tissue collection. Sterile water was administered to the CON and ABX dams by gavage. Housing conditions, time of tissue collection, and sample size calculations were performed as described previously.

For experimental intervention with propionate in the context of gut microbiota dysbiosis, treatment procedures for the ABX and CON groups remained unchanged. In the ABX^Pro^ group, female mice underwent the aforementioned ABX treatment; starting from E0.5, they were provided with ad libitum access to a solution of sodium propionate (Sigma‐Aldrich) at a concentration of 200 mmol/L until E13.5 or E18.5, at which point the dams were killed and tissue collection was carried out. CON and ABX dams were provided with sterile water *at libitum*. Housing conditions, time of tissue collection, and sample size calculations were performed as described previously.

### Water avoidance stress (WAS)

The WAS paradigm was conducted by placing the offspring from the WAS group on a platform (3 × 6 cm) elevated 10 cm above the ground and fixed in the center of a box (60 × 50 cm) filled with room‐temperature water, with the water level reaching 1 cm below the top of the platform. The sham group was not subjected to water filling, whereas all other conditions remained the same. Each mouse in the WAS group remained on the platform for 1 h at a fixed time in the afternoon for 10 consecutive days. During the modeling period, no interventions were made to the behavior of the mice, and measures were taken to minimize noise, intense light, and other stimuli. At the end of each WAS or sham session, all fecal pellets in the collection container were counted to measure colonic motility [47].

### Fecal water content

The experiment was conducted on the day of the final WAS session. The mice were individually placed in dry bedding‐free cages for 1 h. Fecal pellets were collected every 15 min and immediately covered with centrifuge tube caps. After 1 h, the collected fecal pellets were weighed. Subsequently, the fecal pellets were dried in an oven at 60°C for 24 h and weighed again to determine the dry weight. Fecal water content was calculated using wet and dry weights to determine the proportion of water in the fecal pellets.

### Colonic transit time

The colonic transit speed in mice was assessed using the latent bead test [48]. The experiment was conducted on the second day after the WAS. Briefly, mice were fasted (with access to water) for 12 h. They were then lightly anesthetized using isoflurane (RWD), and a 2.5 mm spherical plastic bead coated with glycerol was gently inserted into the distal colon using a glass rod positioned 2 cm from the anus. Subsequently, the mice were individually placed in bedding‐free cages, and the time from bead insertion to expulsion was recorded. The experiment was repeated twice with a 6‐h interval. The average of the two experiments was calculated as the bead expulsion latency.

### Small intestinal transit time

An experiment was conducted using a bead expulsion test. Each mouse was administered 200 μL of carmine red solution (6% carmine (Macklin) dissolved in a 0.5% carboxymethylcellulose (Bide) water solution via oral gavage. After 45 min, the mice were euthanized by cervical dislocation. The abdominal cavity was opened and the small intestine was carefully removed from the pylorus to the ileocecal junction, minimizing any stretching of the intestinal tract. The small intestine was laid out in a straight line, and the proportion of the distance traveled by the carmine red solution to the total length of the small intestine was measured to assess intestinal transit function.

### Visceral sensitivity test

CRD–EMG was performed according to established methods with some modifications [48]. A latex balloon (1 cm wide, 1.5 cm long) attached to a thin catheter (1 mm inner diameter, 1.5 mm outer diameter) at the head was prepared as a pressure‐sensitive balloon. The mice were fasted (with access to water) for 24 h before visceral sensitivity testing. After light isoflurane anesthesia, a glycerol‐coated balloon was inserted into the rectum of the mouse, positioning its distal end approximately 1 cm from the anus. The catheter connecting the balloon was secured to the base of the mouse tail using adhesive tape. The distal end of the balloon catheter was connected to a pressure gauge (World Precision Instruments) and a syringe via a three‐way stop coat. After a 20‐min adaptation period, the balloon was rapidly inflated at pressures of 20, 40, and 60 mmHg in a random order, with each inflation maintained for 20 s and a 4‐min interval between inflations. Each pressure measurement was repeated thrice. Signals were collected and analyzed using a Biological Signal Acquisition System software (Taimeng). Changes in visceral sensation were represented by subtracting the area under the curve (AUC) for 20 s, corresponding to the pre‐distension baseline, from the AUC for 20 s of distension.

### Histology, electron microscopy, and immunofluorescence staining

Hematoxylin and eosin (H&E) staining was used to assess intestinal mucosal development in mouse pups. The ileum and colon were fixed in 10% formaldehyde–phosphate‐buffered saline (PBS) solution for 24 h, dehydrated, embedded in paraffin, and sectioned into 5 μm‐thick slices. After deparaffinization and hydration, sections were stained with H&E (Sigma‐Aldrich), mounted with neutral gum (ZSGB–BIO), and promptly imaged.

Periodic acid‐Schiff (PAS) staining was performed to evaluate the development of goblet cells in the colons of the offspring. Paraffin‐embedded colon sections were deparaffinized and incubated in 1% periodic acid solution (Sigma‐Aldrich) for 10 min. Subsequently, the sections were stained with Schiff's reagent (Sigma‐Aldrich) for 40 min and counterstained with Harris hematoxylin solution (Sigma‐Aldrich) for 25 min. After each step, the stained sections were rinsed with PBS. Finally, sections were mounted with neutral gum and immediately imaged.

For transmission electron microscopy (TEM), colon slices measuring 3 × 3 mm were fixed in 4% paraformaldehyde for 24 h, followed by fixation with 1% osmium tetroxide. The tissues were embedded in neutral resin and sectioned into thin slices. The sections were stained with uranyl acetate and lead citrate, and then stored under dry conditions for imaging purposes, which were promptly conducted.

For whole‐mount staining of the longitudinal muscle‐containing myenteric plexus (LMMP), colon tissue was opened along the mesentery and fixed with a 4% paraformaldehyde solution in a culture dish containing silica gel, with the serosal side facing down. Fixation was performed at 4°C for no longer than 6 h. Then, a 20% sucrose solution (Aladdin) was added for dehydration, and the samples were stored at 4°C until further processing. Subsequently, under a dissecting microscope, the samples were dissected by gently scraping off the mucosa and separating the submucosal and circular muscle layers to obtain the LMMP. The samples were then incubated at room temperature for 2 h in a blocking solution containing 0.3% Triton X‐100 (Aladdin, Shanghai, China) and 10% donkey serum (Solarbio) in PBS. Following blocking, the primary antibody was added and incubated at 4°C for 48 h. After removing the primary antibody solution, the samples were washed thrice with PBS (three times, 15 min each) and incubated with the secondary antibody at room temperature for 4 h. Subsequently, the samples were co‐incubated with Hochest33342 (Solarbio) for 5 min to label the cell nuclei, followed by PBS washes (five times, 10 min each). Whole mounts were placed on adhesive‐coated glass slides, covered with a fluorescent mounting medium, and stored in the dark at –20°C for further analysis. For whole fetal mouse intestinal nerve mounts, no microdissection steps were required. The primary antibody was incubated at 4°C for 24 h, and the remaining steps were the same as for the myenteric plexus whole mounts. All the antibodies were diluted in PBS containing 0.3% Triton X‐100 and 10% donkey serum.

Immunofluorescence staining of cryosections was performed to assess the number of mast cells in the adult mouse offspring. Intestinal tissue was fixed in 4% paraformaldehyde for 6 h at 4°C. After washing with PBS, the tissue was immersed in a 20% sucrose solution in PBS at 4°C for 24 h, followed by transfer to a 30% sucrose solution at 4°C for another 24 h. Subsequently, the tissue was embedded in optimal cutting temperature compound (SAKURA) and stored at –80°C. Cryosections of 8 μm thickness were cut and mounted on adhesive‐coated glass slides, equilibrated to room temperature, and incubated at room temperature for 1 h for blocking. Then, the primary antibody was added to the blocking solution and incubated overnight at 4°C. The next day, the sections were washed three times with PBS (5 min each) and incubated with secondary antibody at room temperature for 1 h. The sections were then co‐incubated with Hoechst33342 for 5 min to label the cell nuclei, washed with PBS (three times, 5 min each), and finally covered with an anti‐fade mounting medium for fluorescence and stored at –20°C in the dark for further analysis.

Antibodies and their working concentrations are shown in Table S1.

### Image acquisition and analysis

Whole‐slide scanning of H&E and PAS‐stained sections was performed using a NanoZoomer (Hamamatsu). Image acquisition of the E18.5 fetus and offspring ENS staining was performed using a confocal microscope (Zeiss). Fluorescence imaging of mast cells from the E13.5 fetus and adult offspring ENS was performed using an inverted fluorescence microscope (Zeiss). The TEM images of ultrathin sections of adult offspring were acquired using TEM (JEOL) at 120 kV. All samples were exposed to the same equipment for the same duration. To assess postnatal intestinal development, the length and cell count of 20 randomly selected villi or crypts in each animal section were measured. To count postnatal neurons and glial cells, 10 randomly selected ganglia were counted on each slide. To calculate the positive area and positive cell count, 10 images were selected at 20× objective magnification for each sample, and ImageJ software (RRID:SCR_002285) was used to analyze and measure the pixel number or cell count of positive immunolabeled regions of interest. For electron microscopy, at least five fields of view were studied for each sample at a magnification of 5000×. The experimental results were evaluated by an observer who was blinded to the experimental conditions.

### *In vitro* intestinal motility assessment

The in vitro assessment of intestinal motility was performed using a validated method with some modifications [49]. Juvenile or adult mouse colonic segments (approximately 1 cm) were obtained by cervical dislocation after euthanasia. The segments were mounted in an organ bath filled with Krebs buffer solution and continuously introduced with 95% O_2_ and 5% CO_2_ at 37°C for 20 min or until a stable baseline was reached, followed by the application of 0.3 g tension. Muscle contraction and relaxation levels were measured before and after drug treatment using a tension sensor system (Taimeng). Between each drug treatment, the colonic segments were washed thrice with Krebs solution. Each colonic segment was subjected to two tests. During this process, the Krebs solution was changed every 15 min. The following drug concentrations were used: 10 μM carbachol (Aladdin), 50 μM atropine (Aladdin), 2 mM l‐NAME (Macklin), and 15 mM l‐arginine (Macklin). Results were calculated as g tension/g dry tissue weight.

### Quantitative real‐time polymerase chain reaction (qRT‐PCR)

qRT‐PCR was performed to analyze the total RNA extracted from embryonic or offspring colons using a Tiangen RNA Extraction Kit (TIANGEN). Following the determination of the RNA concentration, cDNA amplification was performed using a cDNA synthesis kit (Absin). The cDNA, qPCR mix (Toyobo), and the corresponding primers were mixed and subjected to a reaction in QuantStudio 5 (Thermo Fisher Scientific). mRNA expression was normalized using *Gapdh* as the reference gene, and the mRNA levels were calculated using the 2^−ΔΔCt^ method. The primer sequences are listed in Table S2.

### Transcriptome sequencing

The dams in the CON and ABX groups were euthanized at E18.5 via cervical dislocation, and the colons were dissected from their embryos. Total RNA was extracted using the Tiangen RNA Extraction Kit. Subsequently, cDNA amplification was performed using a cDNA synthesis kit, followed by the construction of an mRNA library. After initial library construction, the concentration of the library was detected using a fluorescence quantifier (Qubit 4.0), and the fragment size was assessed using a Qsep400 high‐throughput biofragment analyzer. Finally, the effective concentration of the library was quantified using qPCR. Sequencing was performed by Metware Biotechnology Co., Ltd. using a NovaSeq. 6000 platform (Illumina) to generate 150‐bp paired‐end reads. Fastp v0.23.2 and HiSAT2 v2.2.1 were used for quality filtering and sequence alignment, respectively. Differential expression analysis was performed using DESeq. 2 v1.38.3. The differentially expressed genes were subjected to GO enrichment analysis using DAVID v2023q4. KEGG pathway enrichment analysis and GSEA for KEGG and GO analyses were performed using clusterProfiler v4.6.0.

### Luminex liquid suspension array chip

A MILLIPLEX Mouse High‐Sensitivity T‐Cell Magnetic Bead Panel kit (Millipore) was used according to the manufacturer's instructions. Briefly, the protein supernatant extracted from the colonic tissues of adult offspring was incubated overnight in a 96‐well plate embedded with microbeads. Subsequently, the plate was incubated with detection antibodies for 1 h. Streptavidin Phycoerythrin was added to each well for 30 min, and the values were read using a calibrated Luminex 200 system (Luminex Corporation).

### Metagenomic sequencing

Total microbial DNA from preconception dams was extracted using the QIAamp PowerFecal Pro DNA Kit (Qiagen), and the DNA concentration was measured. The NEBNext Ultra DNA Library Preparation Kit (NEB) was used to generate sequencing libraries. DNA samples were sonicated to 350 bp, followed by end polishing, A‐tailing, and ligation. Subsequently, sequencing was performed using Novogene (Novogene) on the NovaSeq. 6000 platform.

The quality of the filtered read pairs was assessed using FastQC (V.0.11.8). Bowtie2 (V.2.5.1) [50] was used to remove the host DNA. The remaining high‐quality reads were used for further analyses. All samples were subjected to species‐level analysis using MetaPhlAn4 (V.4.0.6) [51] and mpa (V.Oct22. CHOCOPhlAnSGB.202212) marker gene databases. Functional analysis was performed using HUMAnN3 (V.3.7) [51] in UniRef90 mode. The detected genes were further grouped into gene families and pathways and then sum‐normalized within HUMAnN3.

Alpha diversity was estimated using Shannon and Simpson indices. Beta diversity was analyzed using the Bray–Curtis distance algorithm and visualized by PCoA. These analyses were conducted using the corresponding computations and plotting scripts within the Parallel‐Meta Suite [52]. LEfSe was applied to the relative abundance of species and pathways to identify group‐associated biomarkers [53]. Features with a linear discriminant analysis score >2.0 and *p*‐value <0.05 were considered statistically significant. Spearman's rank correlation test was used to calculate the correlation between the species.

### M650 targeted metabolome analysis

M650 targeted metabolome analysis was conducted following the standardized workflow of APExBIO (APExBIO). In brief, serum samples (50 μL) or cecal contents (50 mg) were mixed with a pre‐cooled solution of methanol/acetonitrile/water (2:2:1, v/v), vortexed, subjected to 30 min of low‐temperature ultrasonication, and then incubated at –20°C for 10 min. After centrifugation at 14,000 *g* and 4°C for 15 min, the supernatant was collected for further analysis. Following the preparation of external and internal standards for 623 metabolites (Table S3), separation was performed using an Agilent 1290 Infinity LC Ultrahigh‐Performance Liquid Chromatography system (UHPLC) (Agilent) with HILIC (2.1 mm × 100 mm × 1.7 μm) and C18 (2.1 mm × 100 mm × 1.7 μm) columns. Mass spectrometry was performed using an AB 6500 QTRAP mass spectrometer (AB SCIEX). The raw MRM data were processed using MultiQuant software (AB SCIEX) for peak extraction, obtaining peak areas, and determining the ratio of the internal standard peak areas. Quantification was performed based on the standard curves. OPLS‐DA and KEGG pathway enrichment analyses were conducted using MetaboAnalyst v6.0.

### Measurement of SCFAs

Four SCFAs (acetate, propionate, butyrate, and valerate) were prepared as standard solutions in water, 15% phosphoric acid, l‐ethyl ether, and internal standards to create ten concentration gradients. Serum samples (50 μL) or cecal contents (50 mg) were mixed with 15% phosphoric acid and a combination of internal standards and ethyl ether. The mixture was centrifuged at 12,000 rpm and 4°C for 10 min to obtain the supernatant. Separation was performed using a Thermo Trace 1300 gas chromatography system (Thermo Fisher Scientific) equipped with an Agilent HP‐INNOWAX capillary column (30 mm × 0.25 mm × 0.25 μm). Mass spectrometry analysis was conducted using a Thermo ISQ 7000 mass spectrometer (Thermo Fisher Scientific). Proteo Wizard v.3.0.8789 software was used to calculate the SCFA content in the samples.

### In vitro cultivation of *L. reuteri*

The concentration of *L. reuteri* was adjusted to 1 × 10^9^ CFU/mL. Subsequently, 1 mL of this *L. reuteri* solution was inoculated into 10 mL of a liquid culture medium containing peptone, lipids, glucose, propanediol, glycerol, and the necessary electrolytes for *L. reuteri* growth (*n* = 3). The cultivation was maintained at 37°C under anaerobic conditions. Supernatants were collected at 0, 3, 6, and 24 h, centrifuged at 12,000 rpm for 5 min, and then the levels of SCFAs were measured.

### Statistical analysis

The data are presented as the mean ± standard deviation (SD). The Shapiro–Wilk and Kolmogorov–Smirnov tests were used to assess the normal distribution of the data. Unpaired two‐tailed Student's *t*‐test or non‐parametric tests (Mann–Whitney *U* test) were used for comparisons between the two groups. For data involving more than two groups, a one‐way analysis of variance (ANOVA) followed by Tukey's multiple comparison test or non‐parametric tests (Kruskal–Wallis test followed by Dunn's multiple comparison test) was performed. A two‐way ANOVA was used for analyses involving two variables, followed by Tukey's or Sidak's multiple comparison test. Correlation analysis was conducted using Spearman's rank correlation test. The specific statistical tests employed for each panel are described in the figure legends. The value of *n* was varied in the experiments and is specified in each figure legend. The bar graphs represent individual biological replicates as single points. Statistical significance was set at *p* < 0.05. Statistical analyses were performed using GraphPad Prism 10 software (GraphPad Software; RRID:SCR_002798).

## AUTHOR CONTRIBUTIONS

**Cunzheng Zhang**: Conceptualization; methodology; software; data curation; investigation; validation; formal analysis; visualization; writing—original draft. **Yuzhu Chen**: Methodology; software; data curation; formal analysis; visualization; writing—original draft; investigation. **Ruqiao Duan**: Methodology; investigation; formal analysis; data curation. **Yiming Zhang**: Data curation; investigation; formal analysis. **Haonan Zheng**: Data curation; investigation; formal analysis. **Jindong Zhang**: Data curation; investigation; formal analysis. **Tao Zhang**: Data curation; investigation; formal analysis. **Jingxian Xu**: Data curation; investigation; formal analysis. **Kailong Li**: Formal analysis; methodology; data curation; investigation. **Fei Pei**: Data curation; formal analysis; visualization; investigation. **Liping Duan**: Conceptualization; methodology; data curation; investigation; formal analysis; supervision; funding acquisition; visualization; project administration; resources; writing—review and editing.

## CONFLICT OF INTEREST STATEMENT

The authors declare no conflicts of interest.

## ETHICS STATEMENT

The ethics application (LA2022657) was approved by the Animal Welfare and Ethics Committee of Peking University.

## Supporting information


**Figure S1.** Study design and workflow.
**Figure S2.** Effects of maternal preconception antibiotics exposure on offspring of different genders in juvenile stages.
**Figure S3.** Adult offspring of preconception antibiotics exposure dams exhibit visceral hypersensitivity, a compromised mucosa barrier, and an increased susceptibility to modeling of water avoidance stress (WAS).
**Figure S4.** Adult offspring of preconception antibiotics exposure dams exhibit alterations in visceral sensation and colonic mucosal ultrastructure.
**Figure S5.** Alterations in gene expression occur within the embryonic colon of offspring from antibiotic exposure (ABX) dams.
**Figure S6.** Preconception exposure to antibiotics alters the gut microbiota composition of the dams throughout gestation.
**Figure S7.** Maternal mice in the antibiotic (ABX) group reveal altered functional pathway profiles of the gut microbiota during gestation.
**Figure S8.** Alteration in the metabolome in both the cecum and serum of dams in the antibiotic (ABX) treatment group.
**Figure S9.** Multi‐omics analysis unveils pivotal functional pathways of maternal gut microbiota.
**Figure S10.** Correlation analysis targets potential metabolites that affect enteric nervous system (ENS) development.
**Figure S11.** Correlation analysis uncovers metabolites underlying the effect of *Limosilactobacillus reuteri* on enteric nervous system (ENS) development.
**Figure S12.** The impact of valerate on enteric nervous system (ENS) development.

**Table S1.** Antibodies and their working concentrations used in this study.
**Table S2.** Primer sequences used in this study.
**Table S3.** Details of the compounds detected by M650 targeted metabolomic analysis.

## Data Availability

The data that support the findings of this study are openly available in NCBI Sequence Read Archive (SRA) at https://www.ncbi.nlm.nih.gov/bioproject/, reference number PRJNA1121950 and PRJNA1121951. Sequencing data were submitted to the NCBI Sequence Read Archive (SRA) database under the PRJNA1121950 (https://www.ncbi.nlm.nih.gov/bioproject/PRJNA1121950) and PRJNA1121951 (https://www.ncbi.nlm.nih.gov/bioproject/PRJNA1121951). The data and scripts used are saved in GitHub https://github.com/Chenyuzhu59/Preconception_ENS_Development. Supplementary materials (figures, tables, graphical abstract, and update materials) may be found in the online DOI or iMeta Science http://www.imeta.science/.
