# Supplementary material for: Preconception maternal gut dysbiosis affects enteric nervous system development and disease susceptibility in offspring via the GPR41–GDNF/RET/SOX10 signaling pathway
Source: Imeta. 2025 Mar 18;4(2):e70012. doi: 10.1002/imt2.70012 (PMC11995169; doi:10.1002/imt2.70012)
Supplement: Supplementary file 1 — Figure S1. Study design and workflow. Figure S2. Effects of maternal preconception antibiotics exposure on offspring of different genders in juvenile stages. Figure S3. Adult offspring of preconception antibiotics exposure dams exhibit visceral hypersensitivity, a compromised mucosa barrier, and an increased susceptibility to modeling of water avoidance stress (WAS). Figure S4. Adult offspring of preconception antibiotics exposure dams exhibit alterations in visceral sensation and colonic mucosal ultrastructure. Figure S5. Alterations in gene expression occur within the embryonic colon of offspring from antibiotic exposure (ABX) dams. Figure S6. Preconception exposure to antibiotics alters the gut microbiota composition of the dams throughout gestation. Figure S7. Maternal mice in the antibiotic (ABX) group reveal altered functional pathway profiles of the gut microbiota during gestation. Figure S8. Alteration in the metabolome in both the cecum and serum of dams in the antibiotic (ABX) treatment group. Figure S9. Multi‐omics analysis unveils pivotal functional pathways of maternal gut microbiota. Figure S10. Correlation analysis targets potential metabolites that affect enteric nervous system (ENS) development. Figure S11. Correlation analysis uncovers metabolites underlying the effect of Limosilactobacillus reuteri on enteric nervous system (ENS) development. Figure S12. The impact of valerate on enteric nervous system (ENS) development. [file IMT2-4-e70012-s002.docx]

# Preconception maternal gut dysbiosis affects enteric nervous system development and disease susceptibility in offspring via the GPR41–GDNF/RET/SOX10 signaling pathway

**Running title**: Preconception microbiota affects enteric nervous system development of offspring

Cunzheng Zhang^1,2,3^, Yuzhu Chen^1,2,3^, Ruqiao Duan^1,2,3^, Yiming Zhang^1,2,3^, Haonan Zheng^1,2,3^, Jindong Zhang^1,2,3^, Tao Zhang^1,2,3^, Jingxian Xu^1,2,3^, Kailong Li^4^, Fei Pei^5^, Liping Duan^1,2,3^*

1. Department of Gastroenterology, Peking University Third Hospital, Beijing 100191, China
2. Beijing Key Laboratory for *Helicobacter pylori* Infection and Upper Gastrointestinal Diseases, Beijing 100191, China
3. PKUMed-Wisbiom Joint Laboratory for Human Microbiome Research, Beijing 100191, China
4. Department of Biochemistry and Biophysics, Beijing Key Laboratory of Protein Posttranslational Modifications and Cell Function, School of Basic Medical Sciences, Peking University, Beijing 100191, China
5. Department of Pathology, Peking University Third Hospital, Beijing 100191, China

*Corresponding Author: Liping Duan, MD., E-mail: duanlp@bjmu.edu.cn,

Mailing address: 49 # Garden Road, Haidian District, Beijing, 100191, China.

### SUPPORTING INFORMATION

### Supplementary figures

**
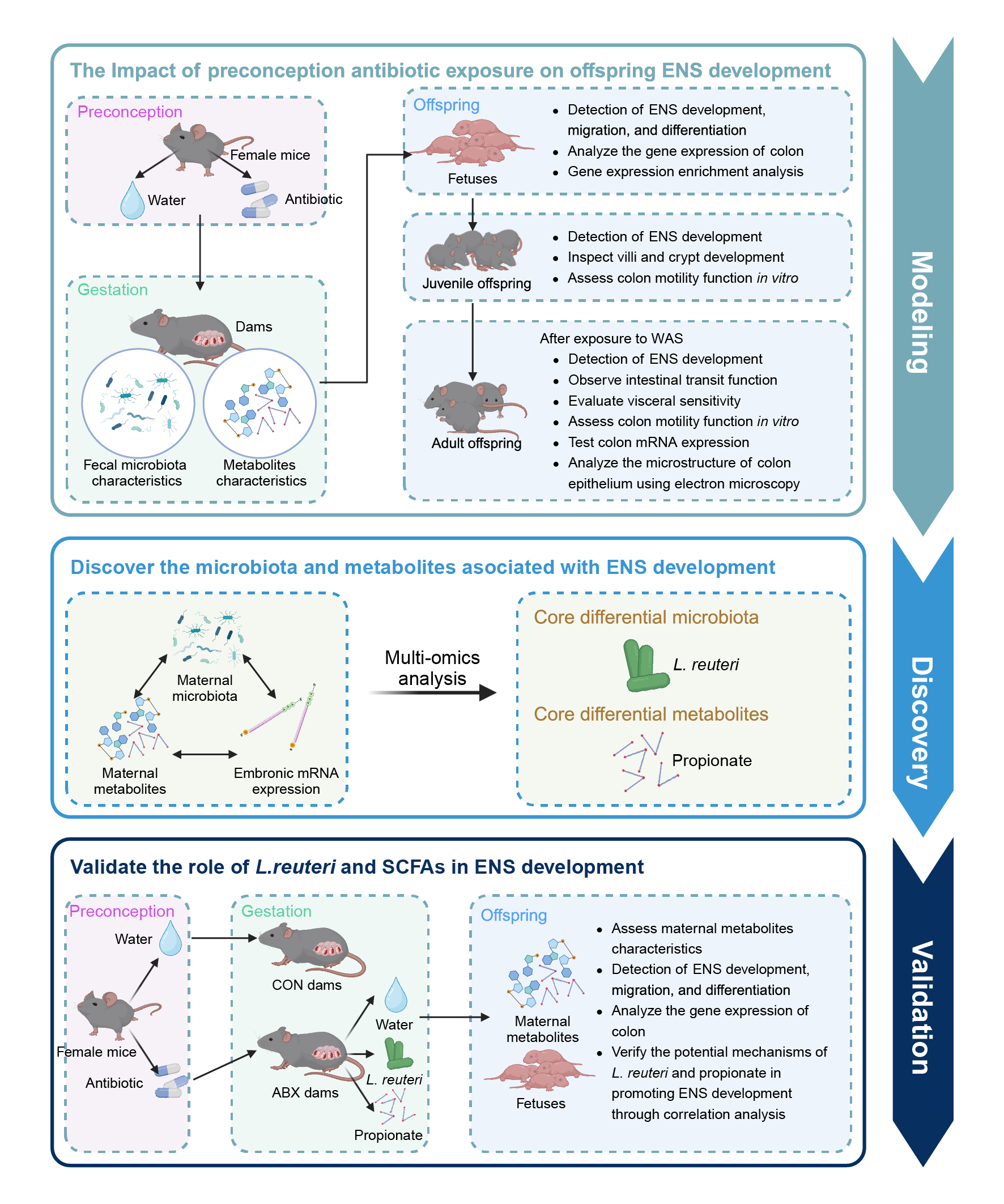
Figure S1. Study design and workflow.** The study initially examined whether maternal preconception gut microbiota disruption affects the development of the ENS in offspring and at which stage this phenomenon occurs. Subsequently, through multi-omics analysis, core maternal microbiota and metabolites that impact ENS development in the offspring were identified. Then, the study employing gestational intervention with *L. reuteri* and propionate to confirm the discoveries and explore their potential mechanisms. ENS, enteric nervous system; CON, control; ABX, antibiotics; SCFAs, short chain fatty acids.

**Figure S2. Effects of maternal preconception antibiotics exposure on offspring of different genders in juvenile stages.** (A). Average body weight of the offspring (*n* = 7–9). (B). Cell counts of HuC/D^+^, S100^+^, ChAT^+^, nNOS^+^, and nestin^+^ cells in male (m) and female (f) juvenile offspring (*n* = 4). (C). Intervention with CCh, atropine, L-NAME, and L-arginine on colonic muscle strips in offspring of different sexes. The contraction or relaxation tension was measured, and the tension per gram of dry weight was calculated (*n* = 2–4) (D). Assessment of villi in the ileum, colonic crypts, and goblet cell count in the colon of offspring of different sexes (*n* = 3–5). Data are presented as mean ± SEM, **p* < 0.05, ***p* < 0.01, ****p* < 0.001 by two-way ANOVA followed by Tukey's multiple comparison test.

**Figure S3. Adult offspring of preconception antibiotics exposure dams exhibit visceral hypersensitivity, a compromised mucosa barrier, and an increased susceptibility to modeling of water avoidance stress (WAS).** (A). Colorectal distension–electromyography (CRD–EMG) in adult offspring at 20 mmHg, 40 mmHg, and 60 mmHg (*n* = 6–7). (B). Representative traces of CRD–EMG in adult offspring at 60 mmHg. (C). Ultrastructural presentation of the colon in adult offspring under electron microscopy. Red arrows indicate microvilli, yellow arrows indicate tight junctions, and white arrows indicate desmosomes (*n* = 3, bar = 500 nm). (D). Relative mRNA expression of occludin in the colon of adult offspring (*n* = 8). (E). Relative mRNA expression of tumor necrosis factor (*Tnf*)-α and colony-stimulating factor (*Csf*)-1 in the colon of adult offspring (*n* = 7–8). (F). Tryptase^+^ mast cell staining and cell count in the colon of adult offspring (*n* = 4, bar = 50 μm). (G). Concentrations of inflammatory cytokines in the colon of adult offspring (*n* = 6–8). Mean ± SEM, **p* < 0.05, ***p* < 0.01, ****p* < 0.001 by two-way ANOVA followed by Tukey's multiple comparison test.

**Figure S4. Adult offspring of preconception antibiotics exposure dams exhibit alterations in visceral sensation and colonic mucosal ultrastructure.** (A). Representative trace of colorectal distension–electromyography (CRD–EMG) in adult offspring at 20 mmHg. (B). Representative trace of CRD–EMG in adult offspring at 40 mmHg. (C). Measurement of colon epithelial microvilli length in adult offspring (*n* = 3). (D). Width, length, and electron-dense area of colon epithelial tight junctions in adult offspring (*n* = 3). Mean ± SEM, statistical analysis performed using two-way ANOVA followed by Tukey's multiple comparison test.


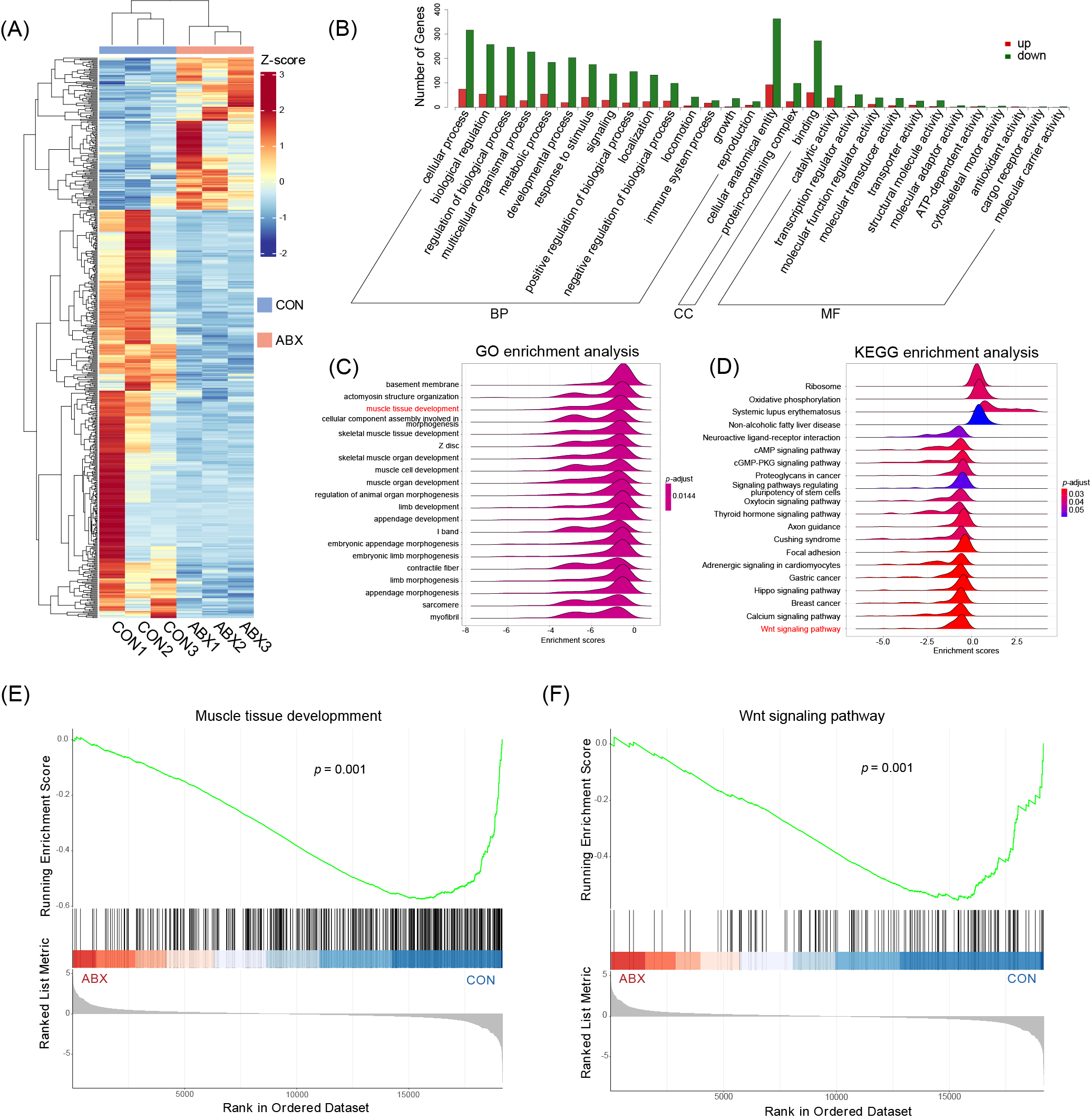


**Figure S5. Alterations in gene expression occur within the embryonic colon of offspring from antibiotic exposure (ABX) dams.** (A). Differential genes were standardized using Z-scores and two-group clustering heatmaps were plotted. (B). Presentation of level 2 Gene Ontology (GO) terms from the GO enrichment analysis (ABX vs. CON). (C). Gene Set Enrichment Analysis (GSEA) using the GO database for the two groups, displaying the 20 entries with the smallest adjusted p-values. (D). GSEA enrichment analysis using the Kyoto Encyclopedia of Genes and Genomes (KEGG) database for the two groups, showing the 20 entries with the smallest adjusted p-values. (E). GSEA enrichment analysis demonstrates the downregulation of "muscle tissue development" in the ABX group. (F). GSEA illustrates the downregulation of the "Wnt signaling pathway" in the ABX group.


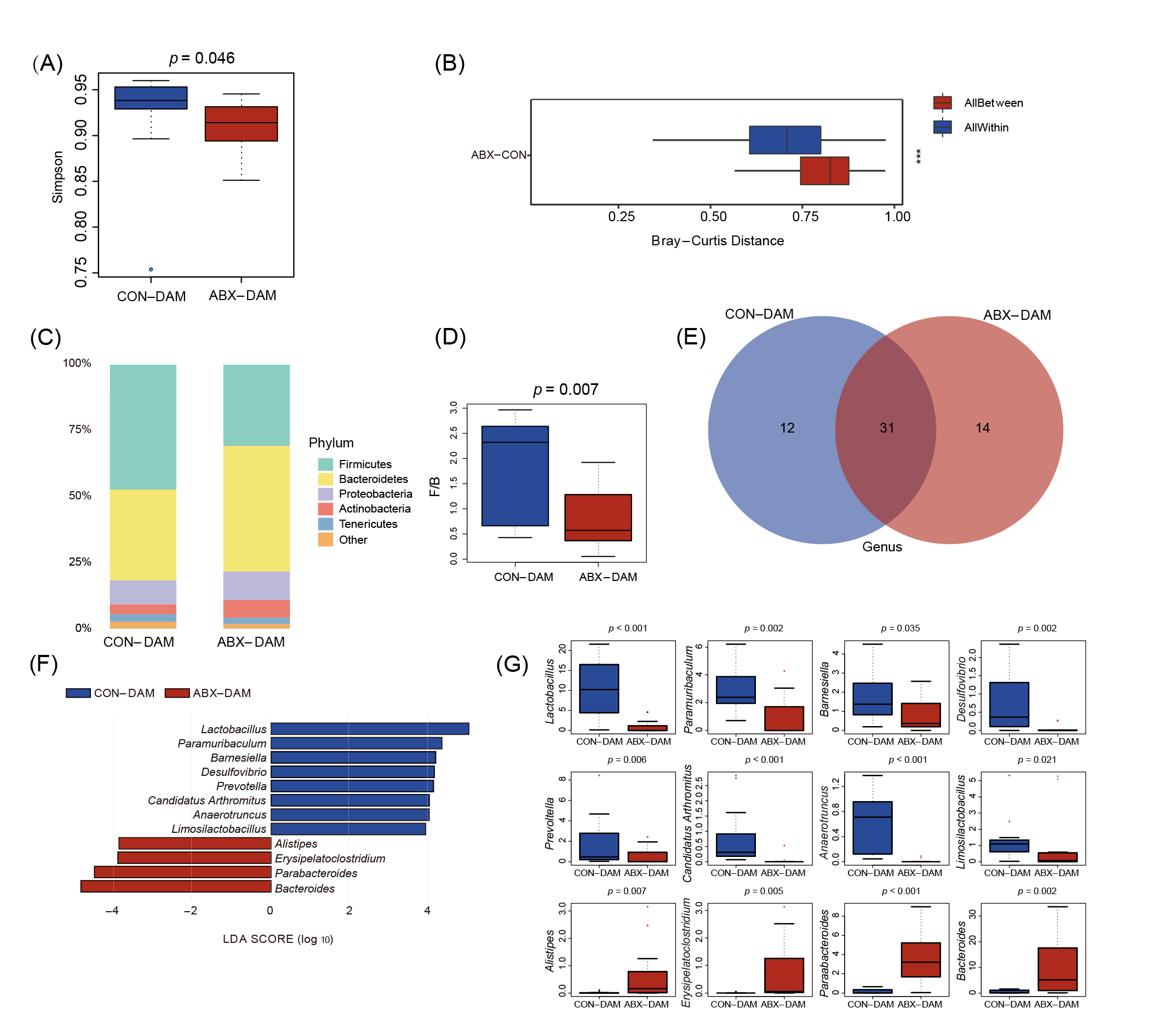


**Figure S6. Preconception exposure to antibiotics alters the gut microbiota composition of the dams throughout gestation.** (A). The Simpson indices of the maternal gut microbiota at the species level on embryonic day 18.5 (E18.5). The differences were calculated using the Wilcoxon test with FDR correction (*n* = 12–13). (B). Bray–Curtis distance of the maternal gut microbiota. (C). Stacked bar graph showing the average abundance of dominant bacterial phyla within each group. (D). Ratio of Bacteroidetes to Firmicutes in the maternal gut microbiota. The differences were computed using the Wilcoxon test with FDR correction (*n* = 12–13). (E). Venn diagram revealing shared and differentially abundant genera between the two groups. (F). Linear discriminant analysis effect size (LEfSe) analysis performed on the 31 shared genera, identifying species with significant differences in abundance between the groups. The differences were computed using the Wilcoxon test with FDR correction. Linear discriminant analysis (LDA) scores were calculated to estimate the effect size of each species' abundance on the observed differences (*n* = 12–13). (G). Display of the average abundance of differentially abundant genera between the two groups.

**Figure S7. Maternal mice in the antibiotic (ABX) group reveal altered functional pathway profiles of the gut microbiota during gestation.** (A)–(B). The Shannon and Simpson indices of the maternal gut microbiota functional pathways profile at embryonic day 18.5 (E18.5) using the MetaCyc database (*n* = 12–13). (C). Principal coordinate analysis (PCoA) analysis of the maternal gut microbiota functional pathways profile using the Bray–Curtis distance. Differences were calculated using the PERMANOVA test (*n* = 12–13). (D). Linear discriminant analysis effect size (LEfSe) analysis performed on the 31 shared genera, identifying species with significant differences in abundance between the groups. The differences were computed using the Wilcoxon test with FDR correction. Linear discriminant analysis (LDA) scores were calculated to estimate the effect size of each species' abundance on the observed differences.

**Figure S8. Alteration in the metabolome in both the cecum and serum of dams in the antibiotic (ABX) treatment group.** (A). Concentration of acetate and butyrate in the cecum of E18.5 maternal mice (*n* = 9). (B). Concentration of acetate and butyrate in the serum of E18.5 maternal mice (*n* = 9). (C). The types and quantity of compounds detected by M650 targeted metabolomics analysis. (D). Orthogonal partial least squares discriminant analysis (OPLS-DA) used to observe differences in cecal metabolites between the two groups of maternal mice (*n* = 9). (E). Correlation plot showing the correlation between differentially abundant metabolites in the cecum of maternal mice. Correlation analysis was performed using the Spearman's rank correlation test, and points with *p* < 0.05 were selected. Larger points indicate smaller p-values. (F). Differential abundance scores of metabolites in the cecum of maternal mice using the Kyoto Encyclopedia of Genes and Genomes (KEGG) database. Larger points at the ends of the lines indicate a higher number of metabolites in that pathway. (G). OPLS-DA was used to observe differences in serum metabolites between the two groups of maternal mice (*n* = 9). (H). Similar to E, a correlation plot showing the correlation between differentially abundant metabolites in the serum of maternal mice. (I). Similar to F, the calculation of differential abundance scores of metabolites in the serum of maternal mice using the KEGG database. **p* < 0.05, ***p* < 0.01, ****p* < 0.001 indicated by Mann–Whitney U test (A, B)

**Figure S9. Multi-omics analysis unveils pivotal functional pathways of maternal gut microbiota.** (A). Heatmaps of differential functional pathway spectra and differential cecal and serum metabolites in E18.5 maternal mice (*n* = 11). Correlation analysis was performed using the Spearman's rank correlation test. Significance levels: **p* < 0.05, ***p* < 0.01, ****p* < 0.005. (B)–(E). Core functional pathways annotated by their taxonomic contributors. The Y-axis represents the relative abundance of MetaCyc pathways, while the X-axis represents dams (*n* = 12–13). Detect p-values using the FDR-corrected Wilcoxon test.

**Figure S10. Correlation analysis targets potential metabolites that affect enteric nervous system (ENS) development.** (A). Scatter plots and linear regression analysis showing the correlation between the concentration of cortexolone and corticosterone in the serum of E18.5 maternal mice and mRNA expression of *Ret, Gdnf*, and *Sox10* in the embryonic gut. Correlation analysis was performed using the Spearman's rank correlation test (*n* = 11). (B). Scatter plots and linear regression analysis showing the correlation between the concentration of selected differentially abundant metabolites in maternal mice and their respective receptors. Correlation analysis was performed using the Spearman's rank correlation test (*n* = 11). (C). Relative mRNA expression of metabolite and bacterial component receptors, including *Tgr5, Tlr2, Tlr4, Nr3c1*, and *Nr3c2*, in the colons of fetal mice. Mean ± SEM, **p* < 0.05, ***p* < 0.01, ****p* < 0.001 by unpaired Student's t-test (*n* = 7–8). (D). Heatmap showing the correlation between the relative mRNA expression of receptors and important genes involved in ENS development in the colons of E18.5 fetal mice. Correlation analysis was performed using the Spearman's rank correlation test. The larger the square and the darker the color, the larger the |r| value. **p* < 0.05, ***p* < 0.01, ****p* < 0.005.

**Figure S11. Correlation analysis uncovers metabolites underlying the effect of *Limosilactobacillus reuteri* on enteric nervous system (ENS) development.** (A). Concentration of butyrate and valerate in the cecum of maternal mice (*n* = 6). (B). Concentration of acetate, propionate, butyrate, and valerate in the cecum of maternal mice (*n* = 6). (C). The levels of acetate, propionate, butyrate and valerate in the supernatant of the *in vitro* cultivation of *L. reuteri* were measured at 0 h, 3 h, 6 h, and 24 h (*n* = 3). (D). Scatter plots and linear regression analysis showing the correlation between the concentration of acetate, butyrate, and valerate in the cecum of E18.5 maternal mice and mRNA expression of *Ret, Gdnf*, and *Sox10* in the embryonic gut (*n* = 18). (E). Scatter plots and linear regression analysis showing the correlation between the concentration of selected differentially abundant metabolites in maternal mice and their respective receptors (*n* = 18). (F). Relative mRNA expression of metabolite and bacterial component receptors, including *Tgr5, Tlr2, Tlr4, Nr3c1*, and *Nr3c2*, in the colons of fetal mice (*n* = 6). (G). Heatmap showing the correlation between the relative mRNA expression of metabolite receptors and important genes involved in ENS development in the colons of E18.5 fetal mice. **p* < 0.05, ***p* < 0.01, ****p* < 0.005. Correlation analysis was performed using the Spearman's rank correlation test (D, E, G). Mean ± SEM, analyzed by one-way ANOVA followed by Tukey's multiple comparison test (F), Kruskal–Wallis test followed by Dunn's multiple comparison test (A, B), or one-way repeated measures ANOVA followed by Tukey's multiple comparison test (C).

**Figure S12. The impact of valerate on enteric nervous system (ENS) development.** (A). The concentrations of acetate, propionate, butyrate, and valerate in the cecum of maternal mice (*n* = 6). (B). Tuj1 and Sox10 staining of the colon in E18.5 fetal mice. The area occupied by Tuj1^+^ neurons and the number of Sox10^+^ cells per mm^2^ were quantified (*n* = 6). (C). Tuj1 staining of the entire intestine of E13.5 fetal mice. Measurements included the length of the entire intestine, width of the colon, the migration distance of Tuj1^+^ cells. The intestine was divided into 10 equal parts, and the proportion of Tuj1^+^ staining in each segment was calculated (*n* = 6). (D). Relative mRNA expression of key genes involved in ENS development in the fetal mouse colon following intervention with propionate (*n* = 6). (E). Relative mRNA expression of short-chain fatty acid SCFA receptors, including *Gpr41, Gpr43*, and *Gpr109A* in the fetal colon (*n* = 6). **p* < 0.05, ***p* < 0.01, ****p* < 0.005. Mean ± SEM, analyzed by one-way ANOVA followed by Tukey's multiple comparison test (B–E) or Kruskal–Wallis test followed by Dunn's multiple comparison test (A).
